# Same-sex competition and sexual conflict expressed through witchcraft accusations

**DOI:** 10.1038/s41598-022-10315-8

**Published:** 2022-04-22

**Authors:** Sarah Peacey, Olympia L. K. Campbell, Ruth Mace

**Affiliations:** Department of Anthropology, University College London, 14 Taviton St, London, WC1E 6BT UK

**Keywords:** Behavioural ecology, Anthropology, Cultural evolution

## Abstract

There is significant cross-cultural variation in the sex of individuals most likely to be accused of practising witchcraft. Allegations of witchcraft might be a mechanism for nullifying competitors so resources they would have used become available to others. In this case, who is targeted may result from patterns of competition and conflict (same-sex or male–female) within specific relationships, which are determined by broader socio-ecological factors. Here we examine patterns of sex-specific accusations in historic cases from sub-Saharan Africa (N = 423 accusations). Male ‘witches’ formed the greater part of our sample, and were mostly accused by male blood-relatives and nonrelatives, often in connection to disputes over wealth and status. Accusations of women were mainly from kin by marriage, and particularly from husbands and co-wives. The most common outcomes were that the accused was forced to move, or suffered reputational damage. Our results suggest that competition underlies accusations and relationship patterns may determine who is liable to be accused.

## Introduction

Witchcraft beliefs were historically prevalent, and are still common in parts of the globe including India, Melanesia and sub-Saharan Africa^1^. Some evolutionary perspectives on such beliefs have suggested they are a result of maladaptive attempts to cure illness^2^ or of psychological predispositions to paranoia and drives to seek explanations for misfortune^3^. Other researchers have suggested that sorcery beliefs level social inequality^4^, promote adherence to social norms^5^, are a means of oppressing women^6^ or act as a social strain gauge to maintain social order^7^, among others. Accusations of ‘witches’ (individuals believed to use supernatural means to harm others) are a significant part of most witchcraft belief systems^6,8^.

Historically and geographically, there is broad variation in the sex thought most likely to practice black magic within societies, despite popular image of witches as female. In modern-day Papua New Guinea (PNG) the gender of accused witches varies by region^9^. A sample of historic small-scale societies found 65% viewed witches as predominantly male or equally likely to be male or female^10^. In parts of Asia and Africa witch-hunts mainly target women^1,11,12^. The causes of this variation are unclear, but it may be a result of socio-ecological factors that determine which sex is more likely to be accused of witchcraft in a culture.

Targeting others through accusations may be an evolutionary mechanism for nullifying competitors^5,11^, as the removal of a ‘witch’ could provide payoffs that contribute to reproductive success. Accusers (and possibly others) gain access to resources and cooperative assistance that would have gone to the accused, at a cost to the latter. Generally in humans, visibly selfish acts towards competitors, where targets are ostracized or physically harmed, would have reputational consequences for actors, decreasing the likelihood that others will cooperate with them in the future^13^. But ‘witches’ represent the antithesis of positive social values^14^. They are typically portrayed as selfish, greedy and intrinsically evil beings, who use their magical abilities to harm others^8,15^. The negative reputational tag of ‘witch’ therefore identifies the accused individual as a ‘cheat,’ so punishment is not only justified but can be portrayed as a public good^3,11,16,17^. Accusations are made harder to refute by the fact that witchcraft acts are hidden and unfalsifiable^4^.

The association between competition and witchcraft allegations is supported by studies indicating accusations increase at times of resource scarcity^18,19^. Generally, societies with greater competition for wealth, such as agriculturalists, have higher levels of witchcraft belief than more materially egalitarian hunter-gatherers^20^. In some cases, competitive gains for accusers are obvious. Ethnographic observers noted that through accusations accusers could reap an increase in status and resources^21,22^, remove rivals^23^, and break ties from costly relationships without sustaining reputational damage^13,24^. That individuals are selected whose ostracism is likely to be profitable is supported by experimental research suggesting those with more resources are more likely to be the targets of mob victimization^25^. Benefits to accusers are not always obvious as accusations have a range of outcomes. Sometimes the accused is gossiped about and avoided^26^, which is still likely to be costly to the ‘witch,’ as they may suffer decreased access to group resources and assistance^11,27^. It may be lower-cost for accusers (who are anonymous and so at less risk of retaliation), and may benefit them by reducing overall levels of competition^28^. There are also accusations which do not appear to cause significant harm, either because they fail to ‘stick’ to targets (that is, they dissipate), or they are resolved through other means such as ceremonies^4^.

If accusations nullify competitors, then the sex of the accused is likely to vary depending on how much competition is directed towards members of a particular sex. Which relationships are most competitive towards one sex or the other will be determined by aspects of socio-ecology: for example kinship structures can have a strong effect on which forms of relationship are competitive and which are cooperative^29^. Theoretical models suggest sex-biased dispersal and descent patterns affect how harming behaviour is directed towards a particular sex^30,31^. Similarly, ethnographers noted that accusations are frequently motivated by aspects of the relationship between accusers and accused, or ‘witches’ and their ‘victims’, in patterns arising from social structure^32^.

Our sample of cases is predominantly drawn from the Bantu ethnolinguistic cluster in sub-Saharan Africa. Therefore it consists of groups with predominantly patrilineal, patrilocal kinship and relatively high levels of polygamous marriage, notwithstanding some variation^33,34^. In patrilineal descent, inheritance is traced through the male line, and in patrilocal residence couples live with the husband’s family after marriage. Here we focus on individual relationships, but we tested predictions for how these broader aspects of socio-ecology might associate with the sex of the accused (see SI Section 3).

We predicted that accusations occurring between unrelated and related individuals are more likely to stem from male-male competition (so both the accuser and the accused will be male). This is because male–male competition is likely to be more intense within these relationship categories than female–female competition or inter-sexual conflict. Since males, unlike females, can increase their fitness by having more partners (and so more offspring), competition between men for mates and resources to attract women can be intense^35^. Such effects may be particularly pronounced in societies with polygyny and male-focused kinship systems^31^. First, polygynous marriage systems increase male competition for partners as the number of eligible women for marriages is reduced by some men having more wives^36,37^. Unrelated males thus compete for material wealth, status and political positions that can enhance reproductive success at the expense of other males. Second, patrilineal inheritance and brideprice can mean related males compete with one another for inheritance to marry. Brideprice, a transfer of wealth from the husband’s family to the bride’s, is a prerequisite for marriage and men may require assistance from their male relatives to obtain it. Further assets are required for the ongoing support of wives and offspring^31^. Some pairings of male relatives may be more likely to come into conflict for the family inheritance, such as brother-brother or father-son in patrilineal societies^31^. In some matrilineal societies, where inheritance is through the female line (and which is the next most common form of descent in our sample), wealth may pass from uncle to nephew, or to other male heirs meaning that male relatives also compete with one another for resources^33,34^.

We predicted that accusations of women were likely to occur both because of female–female competition and male–female conflict. Conflict between co-wives for reproductive resources and as a result of differential fertility and child mortality can be a cause of witchcraft accusations^38^, so this would be likely among our sample where polygyny is common.

Sexual conflict within marriage may also lead to accusations of women. Coercive male behaviours towards females enables males to maximize their fitness, for example by preventing partners from leaving relationships, or being unfaithful (to increase paternity certainty), or by generally enhancing male control of the relationship^35,39–41^. Accusations could also make it easier for husbands to dismiss unwanted wives without damaging their own reputations.

Accusations of women might also come from other categories of affinal kin, particularly in patrilocal societies where their relatedness to their affines is low^33,34^. Wives may have to compete with their husband’s kin for his investment and resources. The absence of pre-existing ties and low status and low relatedness to the group as newcomers might increase their vulnerability, particularly before the arrival of children^42^.

Age may be a factor in accusations, as elderly women are often reported to be disproportionately victimized^1,14^. In evolutionary terms, an elderly woman’s post-reproductive status may mean that she provides fewer inclusive fitness benefits to her kin, if she is unable to produce resources but continues to consume them. By associating her with witchcraft, relatives or others supporting her can portray themselves as justified in ending the relationship. Men may be relatively less vulnerable to age-related accusations due to their status increasing with age.

To further examine these patterns, we explored the nature of the competition associated with sex-bias in accusations. Identification of a ‘witch’ is frequently preceded by conflict, such as rivalry for headmanships, inheritance disputes, or quarrels with an unfaithful partner^32^. In line with our predictions concerning relationship patterns, men might be accused more in situations involving the acquisition of material wealth and status. Women on the other hand might be accused more in situations directly connected to fertility, such as in arguments between co-wives. Both sexes could be equally vulnerable to accusations because of interpersonal factors, such as generally having a bad reputation, or arguments that occur from personal insults or slights.

We also examined accusation outcomes, to further investigate what accusers (and perhaps others) might gain from them. We predicted that accusations reduce the ability of a victim to compete for resources.

**Figure 1:**
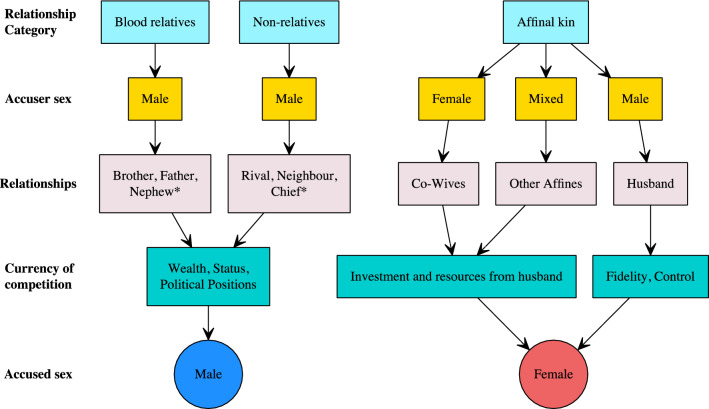
A visual representation of patterns of competition and how they may determine the sex of those accused of witchcraft. *Relationship category* indicates the broad category of relationship that an accusation might stem from. *Accuser sex* indicates the sex of an accuser. *Relationship* gives a more precise specification of the relationship the accuser to the accused (some of the relationship pairings that make up the Relationship Category). Where variables are marked with ‘*’, this means that accusations may stem from other forms of relationship but those given are likely to be common examples. *Currency of competition* indicates what is being contested between the accused and their accuser. *Accused sex* shows whether the accusation is likely to target a man or a woman.

To summarise, our overall hypothesis is that diverse patterns of competition may determine whether accusations target men or women (Fig. 1). To investigate this we used ethnographic accounts to code a variable to examine how three categories of relationship, between blood relatives, unrelated individuals and affinal kin, might predict the sex of the accused. We also had variables for the sex of accusers and the circumstances of accusations. We made the following predictions:

1. Accusations between unrelated individuals are more likely to target men (male–male competition).
2. Accusations between related individuals are more likely to target men (male–male competition).
3. Accusations within three categories of affinal relationship are more likely to target women:
  - Between co-wives (female-female competition).
  - From husbands’ to wives (sexual conflict).
  - From other members of the husband’s kin to the wife (mixed competition).
4. The sex of the accused will vary in relation to the causes of an accusation:
  - Circumstances relating to the acquisition of wealth and status will result in more accusations of men.
  - Situations relating to fertility and relationships will result in more accusations of women.
  - Where circumstances are connected to interpersonal disputes, accusations will be targeted at individuals of both sexes.
5. Accusations of elderly individuals are more likely to target women than men.

## Results

**Figure 2:**
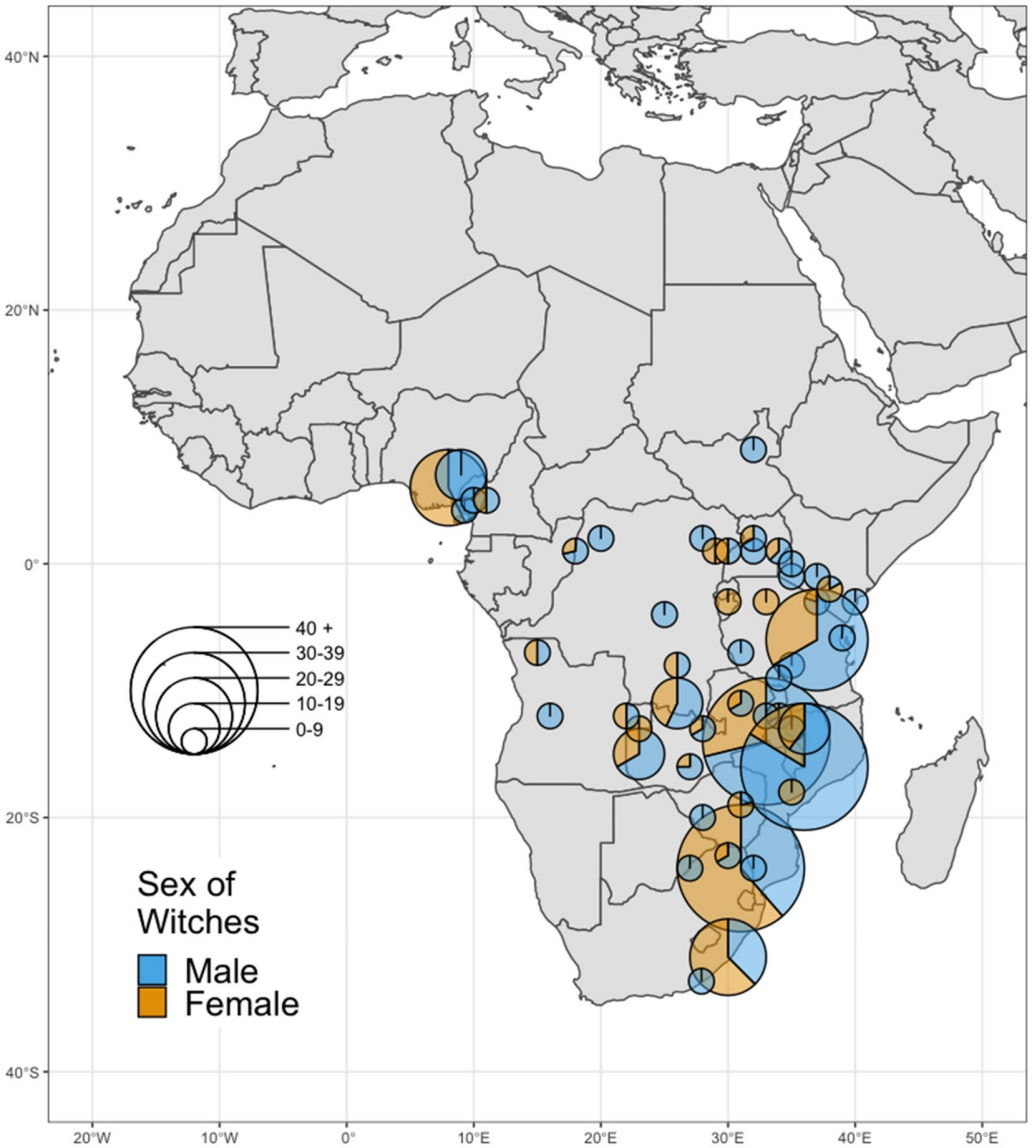
Map showing the location of societies used in the study. Each pie chart represents the percentages of accused men and women making up the total cases in a society. The sample size for each society is illustrated by the size of the pie chart. This was produced using R version 4.0.3^43^ with the packages ggplot2^44^, ggmap^45^ and scatterpie^46^.

More men than women were targeted in accusations (Fig. 2). 37% of ‘witches’ in our sample were female, and 63% were male (see Supplementary Table S1). The intra-class correlation coefficient, calculated using a null model, indicated around 15% of differences in the sex of accused witches was due to differences between societies.

**Figure 3:**
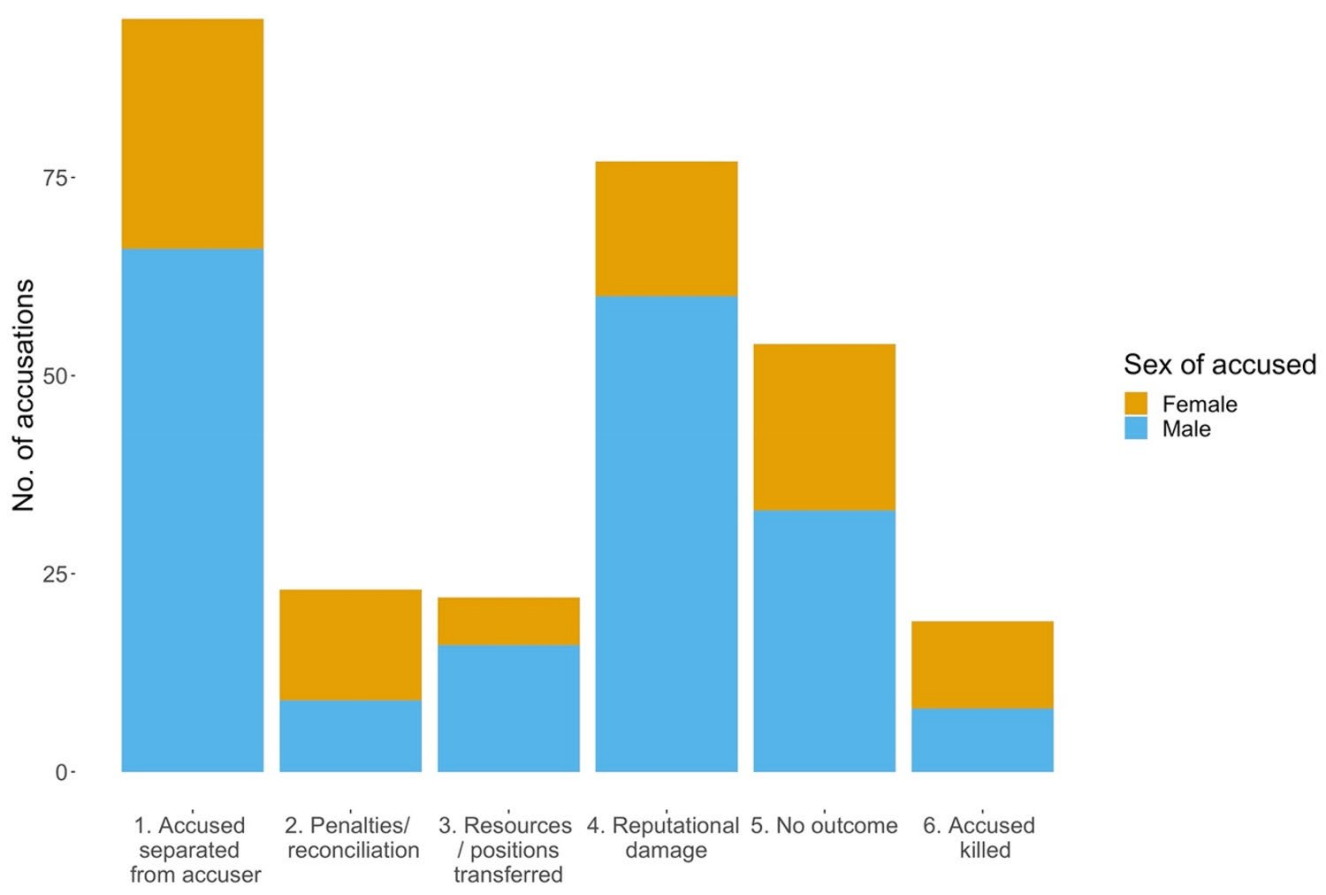
Outcomes from accusations and potential gains by accusers (*N* = 294; NAs are excluded). Columns show counts of how many men and women were subjected to different outcomes when they were accused of witchcraft. 1. ‘Witches’ were exiled from their communities or forced to move away from their accusers. 2. Accused were ‘punished’, e.g., by being beaten, or performing ceremonies to reconcile with accusers, but after that re-instated to communities. 3. Accusers gained directly, receiving material goods or political positions from ‘witches’, or prevented rival ‘witches’ from acquiring them. 4. The main outcome was damage to the reputation of the accused. 5. The accusation did not ‘stick’. 6. The accused was killed.

33% of witchcraft accusations with a known outcome resulted in the accused, whatever their sex, being forced to separate from their accusers (Fig. 3). Reputational damage to the accused was the next most common outcome (27%). Some accusations were dismissed (19%), meaning that they did not ‘stick’ to the reputation of the accused. The true proportion of accusations where this occurred is likely to be considerably higher, but such cases might not have caught the attention of ethnographers. Cases where the accuser directly gained material goods or a political position (8%) were more common when the accused was male. Where the ‘witch’ was ‘punished’ or renounced their witchcraft in a ceremony (8%) they were more often female. 7% of accusations in the sample resulted in the accused ‘witch’ being killed.

**Figure 4:**
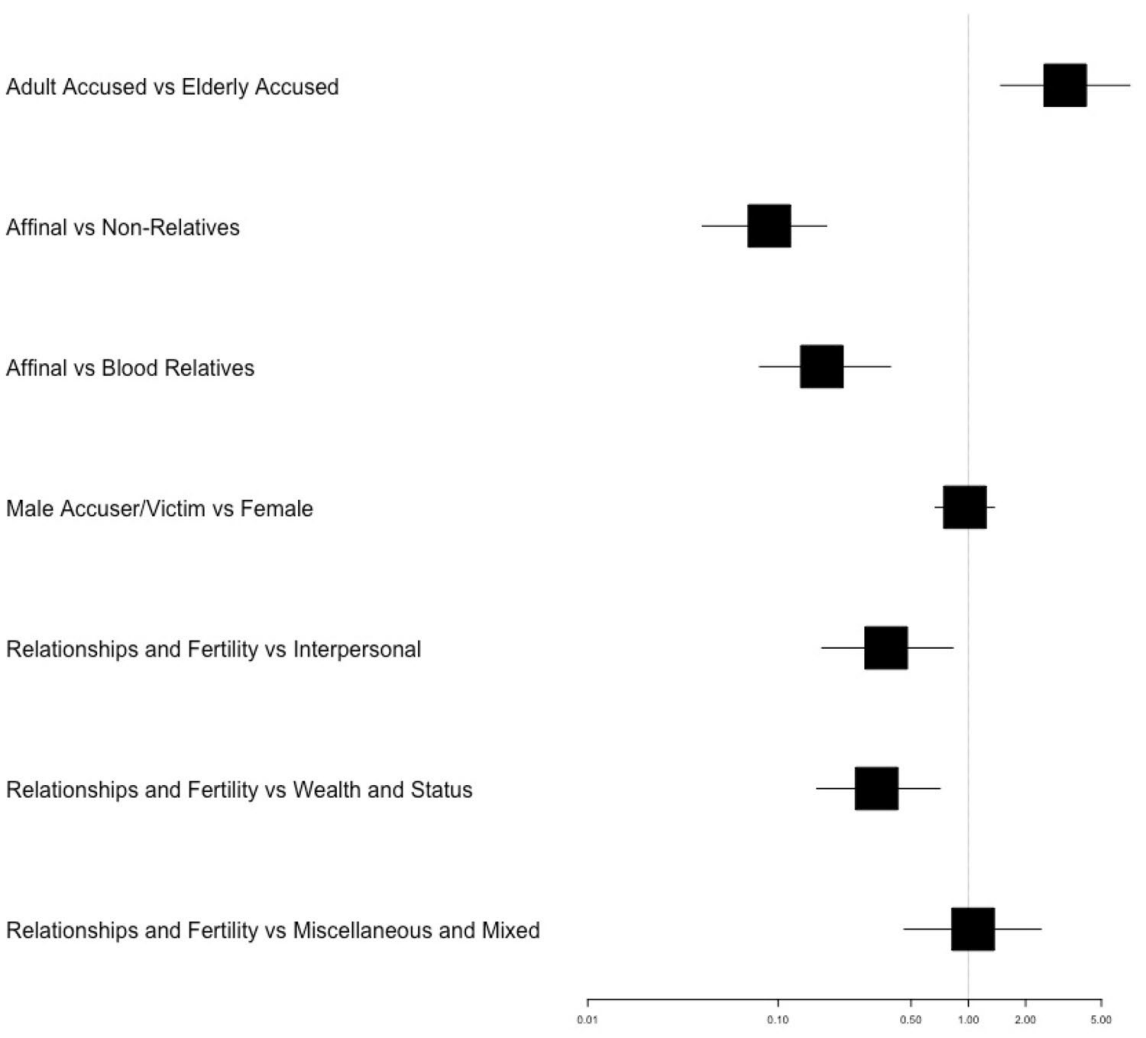
Results from logistic multilevel model-averaging where the outcome variable was the sex of an accused ‘witch’: (0) male or (1) female (*N* = 423). Showing model averaged odds ratios and 95% confidence intervals for predictor variables. These are: (**A**) Age of the accused (0) adult or child and (1) elderly. (**B**) Relationship category of the relationship driving the accusation (which may either be that between the accused and the accuser or between the accused and their purported victim): (0) they are affinal kin (1) they are not related (2) they are blood relatives. C. Sex of the accuser or witch’s purported victim. (0) Male (1) Female. ‘NA’ categories are excluded: full model parameters are provided in the Supplementary Materials (Supplementary Table S3).

The results suggest the category of relationship between accuser and accused can predict the sex of the accused (Fig. 4). Accusations from unrelated individuals, rather than affinal kin, had 11 times higher odds of being directed at men than women (OR 0.09, 95% CI [0.04, 0.18]). The odds that an accusation from a blood relative would target a man rather than a woman were 6 times higher than one from affinal kin (OR 0.17, 95% CI [0.08, 0.39]). 6% of total cases occurred in the context of husband–wife relationships. Of these, 23% were wives accusing their husbands, and 77% were men accusing their wives. 3% of total cases occurred within co-wife relationships. 59% of accusations directed at women by affinal kin came from those who were neither husbands nor co-wives (see SI Fig. 2).

Age was a salient factor in predicting the sex of an accused sorcerer. The odds were 3.23 times higher that an accusation targeting an elderly individual, rather than an adult or a child, would be directed at a woman rather than a man (95% CI [1.48, 7.06]).

All other factors being equal, the sex of accusers or ‘victims’ did not predict the sex of the accused. When an accuser or ‘victim’ was female, the odds of the target of an accusation being female were equal to that of them being male, as when the accuser or ‘victim’ was a man (OR 0.96, 95% CI [0.67, 1.37]). There was a difference in the number of accusations made by each sex: where the sex of accusers was known 19% were from women and 81% were from men.

**Figure 5:**
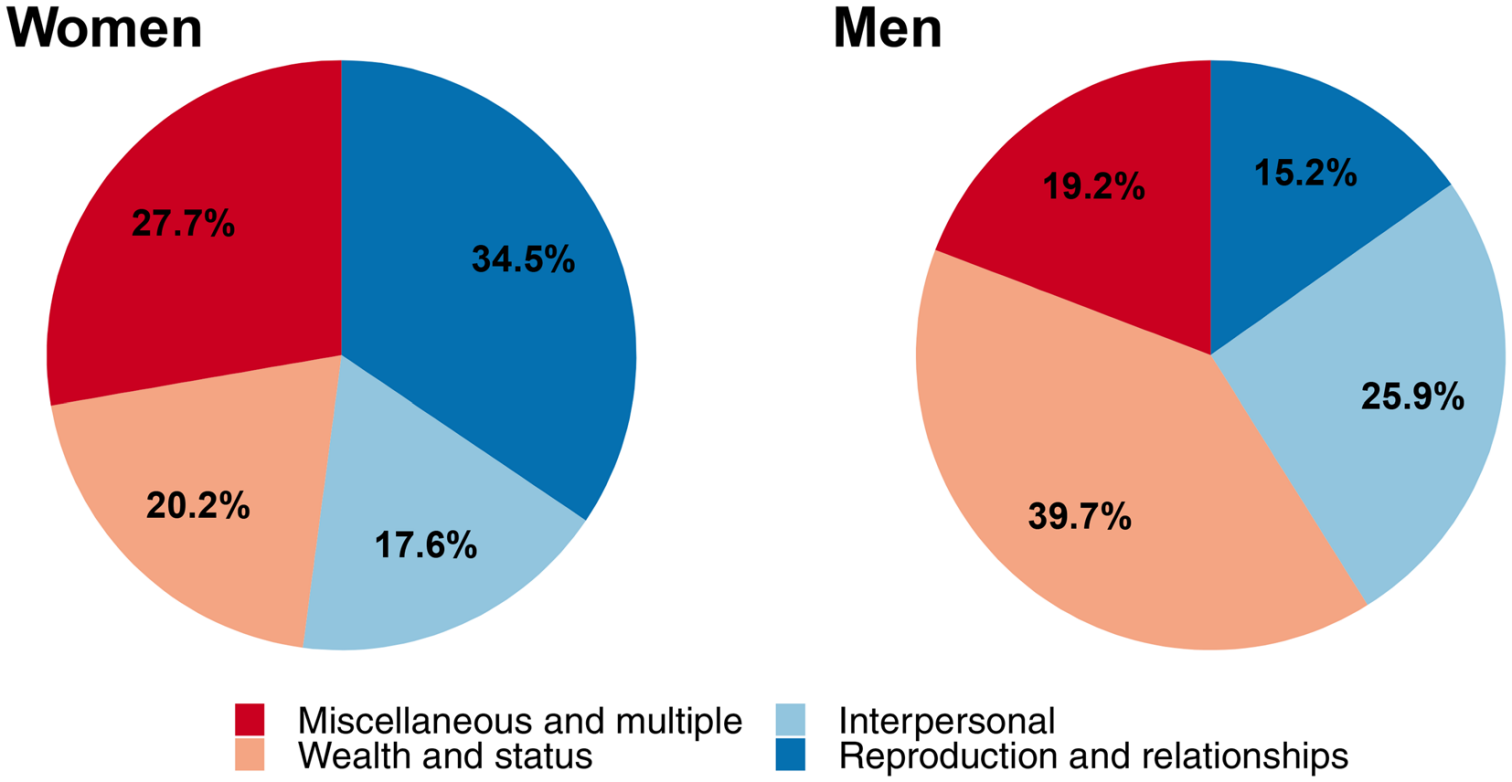
Pie chart showing how ethnographers depicted causes of accusations by sex. The categories are *Reproduction and relationships:* fertility problems, marriage, love, jealousy and adultery. *Wealth and status:* competition for political positions, disputes over property and inheritance, financial/political success. *Interpersonal reasons:* cases where the accused was viewed as difficult, arguments unrelated to previous categories and associated with personal slights, prior reputation as a ‘witch.’ *Miscellaneous cases or Multiple causes.* This category consists of (**a**) cases with no clear reason for a particular individual being identified as a witch; perhaps sometimes solely an explanation for an unfortunate event and (**b**) cases where the causes of an accusation were a combination of the main categories.

The causes or situations relating to accusations, as they were recorded by ethnographers, related more to conflicts over status and wealth when males were accused and reproductive conflict or relationship disputes when women were accused. 40% of cases targeting men were attributed to competition, disputes over property and inheritance (including that of political positions) or envy of success (Figs. 4, 5). In such situations, as compared to accusations that were perceived to stem from fertility or relationship conflicts, the odds of the target being male were significantly higher (inverse OR 3.03, 95% CI [1.41, 6.25]) than that of them being female. Accusations in the ‘Interpersonal’ category also had significantly higher odds of being directed at a man rather than a woman, as compared to accusations relating to fertility or relationships (inverse OR 2.70, 95% CI [1.20, 5.88]). Accusations that apparently resulted from miscellaneous or mixed causes (parts (a) and (b) Fig. 5) were not significantly more likely to target individuals of either sex, compared to accusations associated with relationships and fertility (OR 1.06, 95% CI [0.46, 2.41]).

### Sensitivity analyses

We conducted sensitivity analyses to test for ethnographer bias. These showed that where cases were documented more systematically, and where ethnographers were female, results were largely equivalent to those using the total sample in relation to the sex of accused witches, the sex of accusers and the significant relationships involved in accusations. Where the accused was elderly, sample sizes were reduced, meaning it was not possible to obtain reliable estimates. The variable showing the situation connected to accusations was also mostly no longer significant. Here the result may indicate that the categorisations are somewhat unreliable and results should be treated with caution. However, the sample sizes were also reduced for this variable meaning that there was greater uncertainty in “Results”. The full analyses are given in the supplementary material (Supplementary Tables S8–S11).

### Ethnography meta-data

Recording meta-data on how ethnographic materials were produced could assist in identifying potential biases in observations that would influence results^47^. We examined how factors such as the length of time ethnographers spent doing fieldwork, how fluent they were in the language, the date of publication, the primary role of the ethnographer (e.g., anthropologist or missionary) and the ethnographers’ nationality might influence their observations. The results of this indicated that there were more significant correlations between features of the ethnographies and the results of the ‘situation’ variable than with the other main predictors in our analysis. This suggests that the results of this variable should be treated with caution and may have been more subject to bias than other predictors. Other factors also had an impact on results. The self-reported linguistic knowledge of ethnographers had an effect for some categories within the variables (SI Tables S12–15). This may be because those whose knowledge of the relevant language was only partial were more reliant on their own preconceptions and inferences than those whose fluency allowed them a greater understanding of situations.

## Discussion

The data used here provides evidence that particular relationships may determine sex-specific patterns of witchcraft accusation. Cases where women were targeted frequently came from affinal kin, while those directed at men were often from unrelated individuals and blood relatives. Most previous research on factors that determine the sex of accused ‘witches’ has largely consisted of qualitative studies of a single society or a few societies^48^, or historical studies that have not tested for correlations^49^. Our findings, in support of the overarching hypothesis that accusations may be driven by various forms of competition, can be tentatively aligned with evolutionary literature on patterns of intrasexual and kin competition, intersexual conflict and polygamous mating^30,31,50^.

Men were more often accused than women in our sample, although we did not have a prediction in relation to this. But the finding suggests how overall patterns of competition within relationships may contribute to societal ‘phenotypes’ of witches as male or female. The ethnography of the Ndembele perhaps indicates why women were less frequently targeted in Bantu societies: ‘in a case of witchcraft, the complainant is actuated by caprice, jealousy or pique; and the defendant is a person of wealth or popularity, and is always a man, for the women have neither wealth nor honor worth coveting’^51^.

Our predictions about how the sex of accused ‘witches’ might be associated with particular relationship categories were supported. The majority of accusations targeting men came from unrelated individuals, which is unsurprising, as inclusive fitness^52^ would not mitigate the effects of competition between them. Blood relatives were the next most common relationship category directing accusations at men. This aligns with more recent studies indicating that witchcraft fears between family members are significant in parts of Africa, to the extent that they can be construed as ‘the dark side of kinship’^53^. In evolutionary terms, kin may compete with one another in environments where resources are limited^30,31,50^ and in societies with patrilineal inheritance related males, and particularly brothers, compete for resources in order to marry^31^. This aligns with an ethnographic observation that among the Banyoro witchcraft accusations often occurred between brothers over inheritance, but not between brothers and sisters, whose interests did not conflict^21^. The situations relating to accusations of men were also often connected to the acquisition of wealth and status, such as rivalry over village headmanships^32^, power struggles between a chief’s counsellors^54^ or disputes over inheritance^55^. These connections can be found in more recent contexts such as twentieth century Ghana, where notions of obtaining political power and wealth through occult means involving human sacrifice were pervasive^56^.

Accusations of women were more likely to come from affines. Husbands were the largest category of affinal kin to accuse women (Supplementary Fig. 2). The higher rate of accusations from husbands to wives than wives to husbands aligns with evolutionary perspectives suggesting male coercion of females is a strategy to maximize male reproductive success^39,41^. Accusations of wives who were suspected of being unfaithful can be interpreted as a strategy for reducing investment in unrelated offspring^35,41^. In a case from the Shona a woman gave birth to a stillborn child. This was attributed to an affair before marriage, and was followed by divorce and the repayment of bridewealth to her husband, who commented she was ‘a witch, a woman who had killed her own child’^48^. Other ethnographic accounts suggest accusations of wives by husbands were an attempt to gain control within the marital relationship^55^.

A significant number of accusations of women by affinal kin were from co-wives in polygynous marriages, and these were often notably associated with jealousy connected to a husband’s attention and investment^32^. Evolutionary models predict competition for reproductive resources would occur among co-resident breeding women^57^, as has been found to occur among the Mosuo of southwest China^58^. In the patrilocal social systems that are predominant in our sample, women disperse at marriage and are isolated from kin, so conflict may be more extreme^30^. This is consistent with ethnographic observations reporting that the relationship between co-wives in polygynous marriages was often (although not always) marked by conflict, and liable to produce witchcraft accusations^38,59^.

There were accusations of women from other categories of their affinal kin (Supplementary Fig. 2). These again may result from competition for a husband’s time and resources between his kin and wife. New wives may be vulnerable in environments where they enter their husband’s families as unrelated strangers, and are potentially expendable, at least before the arrival of offspring. Some accounts of accusations indicate that accusations of wives by in-laws in patrilocal households are common^29^.

Accusations directed at elderly individuals targeted women more often than men. This may form part of a broader pattern of geronticide: societies close to subsistence-level are documented as sometimes accepting the abandonment or killing of elderly people^19,60^. In modern Tanzania, ‘witches’ are mostly post-reproductive women, who are more likely to be murdered in periods of income shock^19^. This is also the case in contemporary Ghana, where accusations are frequently directed at middle-aged or elderly women, whose families may subsequently cease to provide them with financial or material assistance^61^. In our sample, elderly women may have been targeted more frequently as a result of longer female lifespans: in a polygynous society, men may marry younger women, so wives would be widowed at an earlier age than husbands. Among the Bantu, older men were accused, but some were possibly protected by their status.

Accusers’ payoffs from accusations are not always explicit but they can be inferred. The most common outcome of accusations in our sample was that accused ‘witches’ were exiled from their communities or forced to move from where they were living. This would mean resources and cooperative assistance they would have used became available to their accusers or others nearby. Where the accused acquires a negative reputation, which was the second most common outcome, there may be a subtle removal of benefits, which may be preferred to direct ‘punishment’ as it is less costly^62^. Accusers’ gains need not be direct, as harming behaviours may reduce the overall pressure of competition in an environment^28^. 8% of accusations in the sample resulted in the acquisition of either resources or political positions from the accused, or in preventing the accused from acquiring them. Where the accused were penalised in other ways, such performing ceremonies to reconcile with accusers, this is perhaps akin to classic cooperation models involving the punishment of defectors (although the accused may not actually be uncooperative)^11^, providing accusers with subordinate partners who offer fitness benefits to avoid more serious allegations^63^. Where an accusation does not ‘stick’, ethnographic accounts sometimes indicate it was reversed through divination or ordeal^54^. In other cases, for various reasons accusations are short-lived and forgotten about^4^. Finally, although not tested in this dataset, accusers may gain informal prestige and dominance, an outcome analogous to competitive punishment^63^.

Not all of the cases in our dataset support the hypothesis that witchcraft accusations are a mechanism for competition. There is a significant proportion where the accusation of a particular individual appears to be incidental, or dependent a on circumstantial association between the ‘witch’ and a negative event. Such accusations are unlikely to provide accusers with a competitive advantage. There are several possible explanations for such cases. They are in line with the hypothesis that witchcraft belief arises from attempts to identify the cause of an impactful misfortune^3,4^. Cultural evolutionary explanations of witchcraft beliefs suggest that they are a maladaptive attempt to explain misfortune. Although it is inaccurate, belief in witches is maintained through bias and selective inattention to evidence that would otherwise counter it^64^. Alternatively this could be viewed under the contention that superstitious beliefs (or errors in attributing cause and effect) are broadly adaptive if they occasionally lead individuals to acts which provide them with fitness benefits^65^.

Although witchcraft accusations may be a mechanism for mitigating the damage to accusers’ reputations in harmful competitive acts, as with any behavioural strategy it is not without risks. Accusers may suffer costs in the form subsequent reputational damage or counter-accusations, as with punishment^63^, depending on factors such the level of support for an accusation by other members of the community.

One limitation of our dataset is that it contains realized allegations of witchcraft, that cannot be tested against baseline population measures. We could not examine the risk that a particular individual, such as an elderly woman, would be accused. Instead, the analysis shows the odds, given an accusation occurred, that the ‘witch’ was male or female, given certain predictors. For example, if the accused was elderly, there are increased odds they were female rather than male.

A dataset using historic witchcraft cases is almost certainly affected by selection bias. Cases with sensational outcomes are more likely to be reported, and cases that are dismissed or where the accused removes themselves from their accusers are liable to be overlooked^19^. Most incidents in our sample were reported anecdotally. Obtaining a random sample of witchcraft accusations within a population is challenging, if not impossible^1,66^. Attempts to systematically collect cases within a given location and timeframe cannot guarantee that all are brought to the attention of researchers^19^. Comparative studies of this kind usually use all the data that is available and control for confounding effects. Our sensitivity analyses suggest the large number of accusations of men in the dataset probably reflects patterns of accusations in these societies, rather than male-focused bias from ethnographers. There are many accounts of cultures where witches are predominantly male^33,34,49^. But the accuracy of historic ethnographic accounts cannot be verified, especially in relation to one-off events such as witchcraft accusations, just as it is unclear how much uncertainty there is in the ethnographic record overall^67^. Ethnographers may not always have noted the characteristics of the individuals involved, or there may be times where they were mistaken in reporting the circumstances surrounding an accusation. There are several explanations for cases where the identities of accusers or purported victims of witchcraft were not reported. Not all cases had identifiable ‘victims’, for example when the accused was thought to have used witchcraft to promote their own success, or ethnographers could not denote the relationship between the accused and their accusers when suspicions of witchcraft were communicated through general gossip. In a small number of cases, ethnographer perspectives on accusations (and possible inability to access further information) are salient, as they may ascribe more importance to one relationship over another in reporting a case, such as a witch’s envy of their victim, or a witch’s argument with an accuser.

However, it is likely that ethnographers were for the most part accurate in documenting variables of interest such as the sex of an accused individual and their relationships with accusers. There is less certainty in relation to the situation connected to an accusation, especially taking into recent research that indicates the prevalence of phenomena such as the misperception of causation^68,69^. Our attempts to account for such possibilities with sensitivity analyses and meta-data on the production of ethnographies cannot conclusively provide reassurance that bias has not affected results, and so this section of the analysis should be treated with caution and regarded as exploratory. The situations documented in our study do however align with accounts of accusations from more contemporary observers and studies from different geographic locations, suggesting that similar causes of accusations arise convergently in different societies. For example in modern contexts accusations have led to accusers gaining land or property in India^6^ and cessation of the obligation to provide material and financial assistance to elderly relatives in Ghana^61^. One advantage of our cross-cultural data being drawn from numerous ethnographies is that it is not reliant on the perspective of one individual, meaning that random perceptual error or individual (as opposed to cultural) bias is more likely to be mitigated in the results than would be the case in the study of a single culture by one ethnographer.

As a further limitation, we were reliant on accessible ethnographic records from the best-documented societies. Although selection bias in favour of better described societies is present in our sample, this should not impact the main aim of this research, which is to understand the determinants of witchcraft accusations being directed at male or female targets.

Overall our findings may indicate allegations of witchcraft stem from diverse forms of competition between individuals. This aligns with evolutionary approaches to competition and conflict. Accusations may provide fitness benefits by allowing individuals to target competitors, but the exact form and direction of competition is determined by aspects of socio-ecology. This in turn influences which sex is most likely to be accused and the overall portrayal of witches in a society. Accusations may be more likely to occur in some relationships rather than others, when there is a gain for the accuser, as in disputes over inheritance and property, or where another individual may pose a threat, or by simply reducing numbers of competitors. The success of witchcraft accusations in removing competitors and their flexibility as an adaptive strategy may explain their widespread distribution.

## Methods

### Study data

The data were collected and coded from 82 ethnographic accounts of historic witchcraft accusations in 54 Bantu and Bantoid societies (*N* = 423) distributed throughout sub-Saharan Africa (Fig. 2). Our sampling frame was taken from Murdock’s Ethnographic Atlas (EA), a large cross-cultural database^34^. Using the EA allowed us to examine accusations from a behavioural ecological perspective, as we acquired a broad cross-cultural sample of accusations which could be analysed for an overview of accusation patterns. The EA also allowed us to use pre-existing variables (see Supplementary Material) and find details of original source materials. The data comes from sources published between 1872 and 1969, when much detailed ethnographic work was conducted and then used to configure the EA, so the main variables and covariates are drawn from a similar period. Our aim was to collect as much data as possible from one region. Randomly sampling accusations from a variety of regions would involve excluding valuable data and would not allow us to compare variation as effectively, especially as there is temporal and spatial variation in how accusations occur^8^ (see Supplementary Material). The Bantu and some of the most closely-related Bantoid societies were chosen as they are a large and particularly well-documented ethnolinguistic family with witchcraft belief^70,71^. They have some variation in patterns of kinship and social organisation yet share common ancestry^33,70^.

We consulted all English-language and some French language ethnographic accounts of the societies that were accessible to us, meaning that we were able to obtain data on all well-documented groups. We reviewed texts for accounts of witchcraft allegations by conducting searches on the Human Relations Area Files (eHRAF) World Cultures website, or reading printed texts sourced from the UCL and British Libraries. In eHRAF we conducted an Advanced Search for each society of interest, using the OR operator with the search terms ‘witch*sorcerer*sorcery*magic*’. Texts that are not part of the EA were for the most part excluded, but we made a small number of exceptions when they matched the EA date range and were particularly relevant. We coded all witchcraft cases we encountered, except those where there was insufficient information on the identity of the alleged witch (especially the sex). We believe that we included almost all cases within our criteria that were available to us. Our main aim was not to estimate the overall prevalence of witchcraft accusations or the frequency with which men or women were targeted, but to examine the determinants of sex-specific accusations.

We confirm that all methods were performed in accordance with the relevant guidelines and regulations. We did not interview or contact any human subjects for this study, and the data used are historic cases that are available in the public domain.

Most societies in the dataset fall into the category of ‘narrow Bantu’ or Bantu proper, with some from closely-related Bantoid groups^72–74^. Societies were identified and categorized in ethnolinguistic terms using several sources: the websites D-PLACE^33^ and glottolog.org^74^, and the articles by Grollemund et al.^70^ and Currie et al.^71^.

References for coding were initially obtained from d-place.org^33^ using societies documented in EA^34^. Additional texts were added when they were relevant and within an appropriate date range.

3 independent coders converted textual accounts of societies and their witchcraft beliefs into categorical variables. These were checked for inter-rater reliability (Supplementary Table S1).

### Variables

#### Accused sex

The outcome variable had two categories: whether those accused were male or female (either individuals or a single-sex group). Ethnographic accounts usually allowed this to be reliably determined. Cases where accusations were directed at individuals of indeterminate sex or groups containing both sexes were classified as ‘NA’.

#### Relationship categories

We recorded the accused individual’s relationship with both their accusers and the purported ‘victim’ of their witchcraft.

In many (but not all) accusations, accuser and ‘victim’ are the same person. Sometimes, the relationship between ‘victim’ and accused is important: if two people have argued and one falls sick, third party observers may claim this is due to the sorcery of the other. In other cases this relationship appears unimportant in the identification of the ‘witch’. For example, if individuals A and B argue, and victim C falls ill, A may claim this was a result of B’s witchcraft.

Each relationship was classified as between blood relatives, affinal kin or unrelated individuals. For some cases (N = 11) a ‘witch’ was accused by people from two relationship categories, in which case the coding refers to the person who initiated the accusation.

Further information on how these variables were coded is included in the Supplementary Information (Supplementary Tables S3, S5–S7).

#### Accuser sex, victim sex

These variables contained the same categories: male/s, female/s. Accusations from groups of both sexes where there was no clear indication of the numbers of each were recoded as ‘NA’.

#### Accused age

This variable indicates whether the accused was reported as ‘old’ or ‘not old’ in the ethnography. There were a small number of cases (*N* = *7*) where the accused was a child or teenager: these were included in the ‘not old’ category.

#### Situations connected to accusations

Accusations usually have more than one cause. Categories here exclude precipitating misfortunes such as sickness or crop failure, which may be (but are not always) separate from the identity of the ‘witch’. The variable records an ethnographer’s depiction of why the accused appears to have been targeted, which is usually connected to social relationships. These may give an indication of why accusations occurred, although there is considerable potential for error or bias on the part of an ethnographer, meaning results from this variable should be treated with caution. Some cases have an ethnographer’s detailed observations of events leading to an accusation or the cause can be inferred from the circumstances. For example, in a case from the Chewa, a dispute occurred concerning the inheritance of a headman’s cattle^32^. The headman’s son, Yolani, was unhappy with his share and following arguments about how many cattle each heir was owed, was reported to have threatened two others with witchcraft as a result. They subsequently died and he was accused of killing them through sorcery. Yolani may have made the threat or it could have been invented as a post hoc justification, but it seems likely (the account is fairly detailed) that this accusation resulted from the disputed inheritance. The situations in these cases are filtered through both the reports of informants and ethnographers, but for many of them there is a description of general circumstances. Despite the need for caution there is no reason to suppose ethnographers did not usually capture the salient aspects of a situation.

The reason an accusation appears to target a particular individual are fairly broad and so most accusations could be placed within one of the categories are given below. (See SI for examples of case studies and further details on coding).

1. Fertility and relationships: cases associated with marital discord, divorce, childbirth, fertility problems and love rivalries.
2. Interpersonal: disputes outside categories (1) and (3), cases where perceived personal traits of the accused (such as aggression) were salient, personal slights and arguments occurred, or where accusations targeted those with a pre-existing reputation for sorcery.
3. Wealth and status: disputes over property and inheritance, competition for political positions, or cases where the ‘success’ of the accused caused accusations.
4. This contains two categories: (A) Miscellaneous: identification of the ‘witch’ appears random, such as when the only factor seems to be the belief that the survivor of a negative event, such as a crocodile attack, must be a ‘witch’. In these cases, the identity of the ‘witch’ appears disassociated from their relationships with others. (B) Cases where the reasons for accusation include more than one of categories 1–3.

#### Outcomes of accusations and gains for accusers

This variable indicates what accusers’ might gain in currencies that translate into fitness benefits.

1. ‘Witches’ were exiled from their communities, or forced to move away from their accusers.
2. Those accused are penalized: they may be beaten or made to renounce their ‘witchcraft’ but remain in their community apparently without further effects.
3. Accusers gain resources or political positions that would have gone to the accused.
4. The accused gains a reputation as a ‘witch’ and is gossiped about or ostracized within their community.
5. The accusation does not ‘stick’ to the accused.
6. The accused was killed. This sometimes occurred as a result of a poison ordeal, a ‘test’ to indicate whether or not the accused was guilty of witchcraft. In other instances the accused was killed through a spontaneous attack or one agreed on by community members.

### Data analysis

We used an information-theoretic model-averaging approach^75–77^ with multi-level logistic models to compare how different variables predicted the sex of accused ‘witches’. Multilevel models were used to take account of variation between societies. All analyses were conducted in R 3.5.3^78^, using the *lme4* package for multilevel modelling^79^ and *MuMIn*^80^ for model averaging.

The model-averaging approach accounts for uncertainty in selecting the ‘best’ model, by averaging parameters across a number of the most plausible models^75,76^. It uses the Akaike Information Criterion (AIC) to examine the relative amount of support for each model, while penalizing inclusion of additional predictors.

The procedure was as follows: (1) specification of a global model containing all variables of interest (2) comparison of all possible subsets of the global model with all possible combinations of predictor variables (3) selection of a ‘top model set’ containing models with the best level of support (with a delta of 6 or lower)^77^, (4), averaging across the top model set to obtain estimates and 95% confidence intervals.

Variables were checked for collinearity using *car*^81^. All variance inflation factors (VIFs) were under 3, indicating a low level of collinearity^82^.

We performed three versions of the analyses, with the sex of the accused as the outcome variable. The first analysis contained variables where categories could be relevant to accuser and the victim, according to which type of relationship with the accused appeared to be driving the accusation. The second analysis used variables relating to accusers. We then repeated the first analysis using society-level variables on descent, post-marital residence and social stratification that could influence whether accused witches were male or female. We used re-classified variables from the EA (SI Section 3); none of the society-level variables had a significant effect on the outcome variable.

We conducted sensitivity analyses to investigate how far our results may have been affected by ethnographers’ reporting biases. These consisted of (1) an analysis using data from societies where ethnographers were as systematic as possible in documenting cases (2) an analysis using case studies collected by female ethnographers to see whether this affected our results (most ethnographic accounts were written by men). (SI Section 5).

## Supplementary Information

Supplementary Information.

## Data Availability

The data associated with this research is available in the UCL Research Data Repository at https://doi.org/10.5522/04/19573198.
