# Supplementary material for: Same-sex competition and sexual conflict expressed through witchcraft accusations
Source: Sci Rep. 2022 Apr 22;12:6655. doi: 10.1038/s41598-022-10315-8 (PMC9033826; doi:10.1038/s41598-022-10315-8)
Supplement: Supplementary file 1 — Supplementary Information. [file 41598_2022_10315_MOESM1_ESM.pdf]

Supplementary Information

Same-sex competition and sexual conflict expressed through witchcraft accusations

Sarah Peacey; Olympia L.K. Campbell; Ruth Mace

CONTENTS

This supplementary material consists of:

1. Societies used in the study: background and why they were selected
2. Materials and Methods: details of variables and how they were coded, including inter-rater reliability
3. Socio-ecology and the sex of accused 'witches': additional predictions
4. Descriptive statistics and full results from individual and society-level variables
5. Sensitivity analyses: A) societies with systematically collected cases and B) cases recorded by female ethnographers
6. Ethnography Meta-Data
7. References used for coding the dataset
8. SI References

1. SOCIETIES USED IN THE STUDY

Our sample consists of witchcraft cases from Bantu and Bantoid societies in sub-Saharan Africa [1]. We began by identifying ethnographies of these societies that are included in the Ethnographic Atlas (EA) [2], as they are listed on the D-PLACE website (www.d-place.org) (4). Each society in the EA has a focal year which the data refer to. These range from the nineteenth to the mid-twentieth centuries, with the majority of cultures being documented in the early twentieth century [3]. The ethnographies we used have focal years ranging from 1850 to 1950 [2,3]. A number of the societies were coded using the Human Relations Area Files World Cultures online database (eHRAF World Cultures: available at https://hraf.yale.edu). We added additional sources, particularly those with more information on witchcraft beliefs and accusations, when they seemed reliable and within an appropriate date range.

We mostly consulted ethnographies that were written in English (see below), but there are a number from French ethnographic records.

The societies in the sample rely mainly on agriculture for subsistence. One society was slightly exceptional for a couple of reasons. The Mbuti speak a Bantu language but are hunter-gatherers rather than agriculturalists and pastoralists. They were not originally part of the Bantu ethno-linguistic group, but probably acquired the language and a low level of witchcraft belief from their Bantu neighbours [4–6]. The one case of witchcraft accusation that was recorded from the Mbuti is included in the dataset [7].

2. MATERIALS AND METHODS

Accusation variables

We coded these variables from ethnographic materials with information on witchcraft cases (see the references below for a full list of sources).

Sex of the accused

0) Male/s
1) Female/s

This was a straightforward variable to code, as the sex of the accused individual was almost always clear from the text. If it was not clear or not mentioned by the ethnographer, the case was excluded from the analysis. The categories here include situations where the accused was a single individual of either sex, and some instances where a group of men or a group of women was accused. When there were several accused witches, cases were generally coded separately for each individual. Groups of men and groups of women were only included as a single case when there was no distinction between those accused, in terms of their identity or relationship to their accusers or 'victims', such as 'a group of men' or 'some women from the village'.

Age of the accused

0) Adult or child
1) Elderly

Many ethnographers only mentioned the age of accused individuals who were particularly old or young, which is why this variable has broad categories. It seems likely these characteristics were recorded because they were notable and could have a bearing on accusations. It was usually apparent when the accused was an adult.

However there may be some cases where ethnographers omitted to record whether an accused ‘sorcerer’ was particularly elderly or not.

Accused-accuser relationship

0) Affinal kin
1) Unrelated
2) Related

The exact relationships between individuals were not always explicitly detailed by ethnographers, although it was usually possible to identify the broader categories given above. Blood relatives include both members of an individual’s nuclear family, and also fairly distant kin, if they are members of the same lineage. Affinal relatives are any people connected to the focal ‘witch’ through marriage, including husbands and wives, co-wives and their children, as well as more distant affinal kin. Unrelated individuals were often neighbours, unrelated political competitors, either the subject or the headman/chief of focal individuals or love rivals.

Accused-victim relationship

0) Affinal kin
1) Unrelated
2) Related

These were categorised in the same way as the relationship between the accused ‘witch’ and their accuser, but using the relationship between the ‘witch’ and their purported ‘victim’.

Sex of the accuser/s

0) Male/s
1) Female/s

This could be slightly more difficult to code than the sex of the accused, as ethnographers sometimes did not view it as relevant, and only gave a name or an initial. Where it was not possible to determine the sex of accusers, it was classified as ‘NA’. There were cases where the accused was targeted by a number of accusers of mixed or indeterminate sex, and these have also been included in the ‘NA’ category.

Sex of the ‘victim/s’

0) Male/s

1) Female/s

This variable refers to the sex of the purported ‘victim’ of witchcraft. As above, this was sometimes more difficult to code than the sex of the accused. Where it could not be determined (for example many of those thought to be targeted by sorcery are infants), it was classified as ‘NA’. There are also instances where ‘victims’ are livestock, rather than humans. Such cases were included under ‘NA’.

Combining ‘victim’ and ‘accuser’ variables

We created variables that combine ‘accusers’ and ‘victims’ because the relationship driving an accusation appeared to vary by case.

A variable (not used in analysis) was coded to show whether the accused ‘witch’s’ relationship with the ‘victim’ was important in an accusation. This was used to select whether the coding for an accuser or a victim was included in the combined variables:

The ‘victim’ is important

0) Yes, the relationship appears to be important in the context of an accusation

1) No, not important.

There are a minority of cases where there was no purported ‘victim’ of witchcraft, in which case the relationship with accusers was automatically included in the combined variables.

The accuser and the ‘victim’ are the same

This was the case for 59% of all cases in the dataset. This relationship was included in the combination variables.

A typical example of this form of accusation is given by Beattie [8] from the Nyoro.

Two women, Keziya and Nyenjura, were married to the same man, Kezironi. Keziya had two children, but Nyenjura had none. Keziya became ill, and went to a diviner who confirmed her suspicions that she was the victim of sorcery by Nyenjura. Keziya’s condition became worse, and the case ended when she assaulted and killed Nyenjura, for which she was prosecuted in the Protectorate courts.

The 'victim' is incidental

Cases where the victim-accused relationship was categorised as unimportant in terms of the accusation. This included all relationships where the 'victim' was an infant or livestock.

Although the victim can be incidental, they are often connected to the accuser. The sickness of a 'victim' often precipitated an accusation by an accuser who already had a grievance against the accused. In other cases accusations may occur because the accuser is looking for an explanation for the illness or death of their relative.

Competition and conflict between the 'victim' and the accused appears more important than the relationship between the 'witch' and their accusers

Jean La Fontaine provided an example of one such case from Bugisu [9]:

Magombe and Zune were age-mates and members of a minor lineage. Magombe lent Zune a cow, and after a lengthy amount of time he asked for it back. He then went to where Zune's cattle were grazing and took the cow back himself. Zune was angry and said 'he would not stand for being treated as though he were an untrustworthy stranger, he a kinsman and an age-mate.'

Some time later Magombe was taken ill. His brothers accused Zune of bewitching him. Zune denied any responsibility for Magombe's illness, but eventually conceded to eat food with Magombe (a way of cancelling witchcraft), after which Magombe recovered.

In the main article, we use the variables where the accuser and 'victim' categories are combined. However, we also conducted the analysis where all variables were between the accused and the accuser, as given below in **Table S5.**

Causes of accusations

0) Fertility and relationships
1) Interpersonal factors
2) Wealth and status
3) Miscellaneous and mixed

As noted in the main text, there are often several concurrent factors involved in a witchcraft accusation. Most cases involve arguments or conflict of some sort. This variable originally had a large number of categories, which were difficult to code in some cases, as it was challenging to reduce situations to a single 'type'. We reduced the categories to those listed above, which has increased the reliability of the code. There were some cases where more than one category remained relevant, which were coded under 'mixed'. We avoided inferring reasons from relationships as much as possible. Although reasons are often stated without ambiguity in ethnographies, they are dependent on the perspective of the ethnographer and their informants, although how much was inferred is likely to vary by case. Some cases are fairly detailed and give the impression that observers were well-informed about the circumstances leading up to an accusation. Others may have been based more on the ethnographers' unique interpretation and therefore less reliable.

Fertility and relationships

This category involves cases where accusations are made because of:

- Arguments and jealousy or inferred jealousy (e.g. where an infertile wife has been assumed to be jealous of a fertile wife and is therefore a witch) between co-wives
- Adultery
- Love rivalry and rejection
- Divorce
- Infertility
- Problems with childbirth

Interpersonal factors

This category includes:

- Cases where the accused was viewed as being difficult, argumentative and uncooperative, or failed to adhere to expected social norms (and this appears to have been their general reputation and pattern of behaviour, rather than having a basis in one-off incidents or a relationship with a particular individual)
- Cases where the accused already has a reputation as a 'witch'
- Arguments that generally stem from personal matters rather than either issues with categories 0) and 3)

- Instances where the ‘witch’ has a trait or disorder, such as epilepsy, which appears to be why they are targeted

Wealth and status

This category includes:

- Cases which result from disputes over inheritance and property
- Cases where the accused and accuser or victim are competing for political positions
- Cases where the ‘witch’ has been successful, or is suspected to have used magic to become successful
- Cases where the witch is thought to have bewitched others because he or she is envious of them
- It also includes a small number of cases where individuals who are vulnerable and reliant on others for resources are accused of witchcraft

Miscellaneous and Mixed

Miscellaneous includes cases where an accusation seems to stem from a need to explain a death or illness, or an otherwise inexplicable event (such as surviving an attack by a crocodile), rather than because of who the accused is in relation to their accusers or ‘victim’.

Mixed cases are those where two or more of the other causes appeared relevant to an accusation.

Table S1. Inter-rater reliability measured by percentage agreement and Fleiss’ Kappa. Ensuring the reliability of data produced from converting text from ethnographic materials into variables for quantitative analysis is crucial. In order to assess the inter-rater reliability of our data, 3 coders independently reviewed a subsample from our overall dataset (43 cases from 3 societies). We used the *irr* package in R [10] to calculate a kappa statistic, which penalises levels of agreement that could be due to chance. For this reason, percentage agreements should also be taken into account, as variables which only have a couple of categories (e.g. ‘Accused Age) may be penalised heavily in kappa calculations. NA variables are automatically excluded [10].

Variable	Percentage agreement	Fleiss’ Kappa
Sex of the accused ‘witch’	95	0.92
Relationship between the accused and the accuser	86.5	0.83
Importance of the relationship between the accused and the victim*	85	0.80
Relationship between the accused and the victim	82	0.81
Sex of accuser	96	0.84

Situation	68	0.65
Accused age	97	0.50

*This variable was not used in the analysis, but was coded to identify the cases where the relationship of the victim and the accused is important in an accusation.

3. SOCIO-ECOLOGY AND THE SEX OF ACCUSED 'WITCHES'

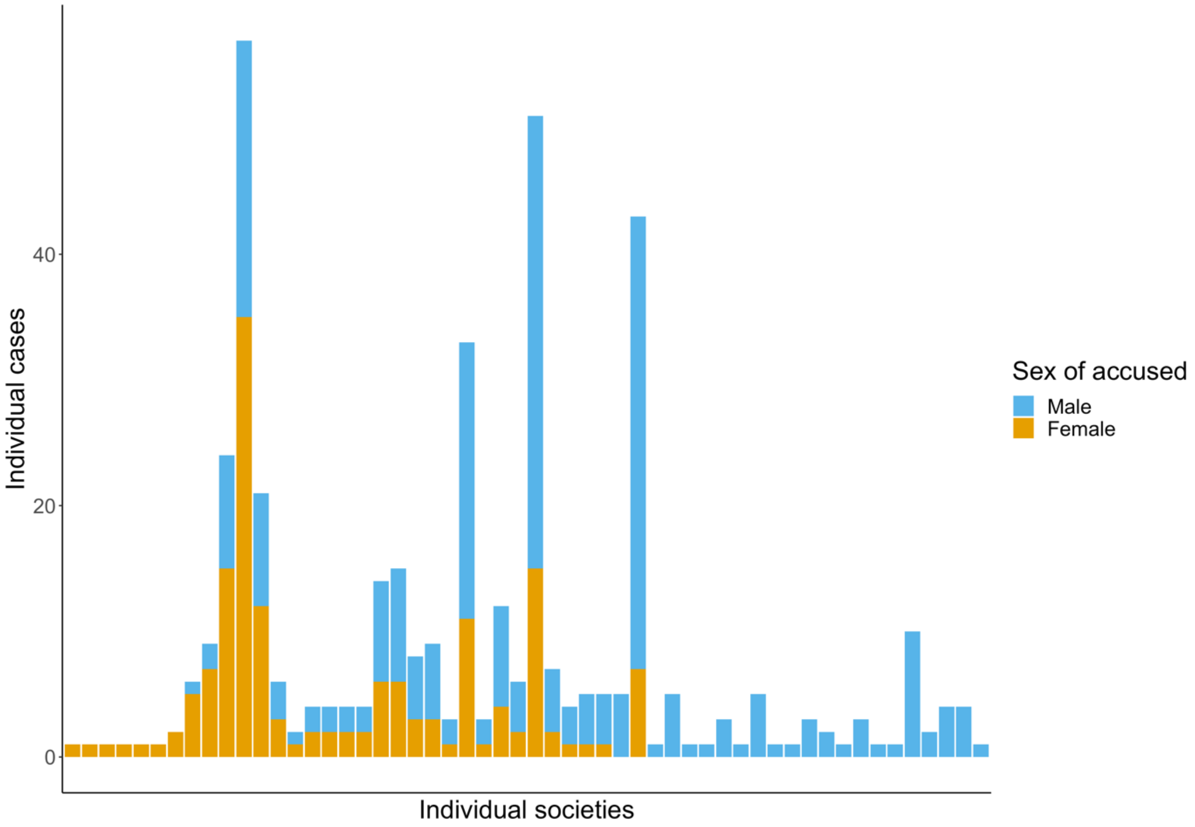

Supplementary Fig. 1. Counts of cases by society, showing the proportion of male and female individuals for each. (Society $N = 54$, Individual $N = 423$). Each column represents one society. They are divided by the sex of the accused, with blue representing men and red representing women. They are ranked from left to right from societies with only female 'witches' to those with both sexes to societies where accusations only targeted men.

We had a number of predictions about socio-ecological factors that may produce higher levels of competition directed at men or women, and subsequently more witchcraft accusations targeting individuals of that sex. These are associated with the question in the main article of why men or women might be more liable to be accused in the context of individual relationships. Some predictions are alternate to one another: for example we predicted that women would be accused more in polygynous societies due to competition for reproductive resources, but it

is also the case that polygyny tends to arise in association with differentiation in male resource distribution, which could lead to more accusations of men [11–15].

3.1 Men are more likely to be accused of witchcraft than women in societies with patrilineal and patrilocal social systems

Where descent and inheritance are traced through the male line, related men (particularly fathers, sons and brothers) are more likely to have to compete intensely with one another in order to acquire the resources they need to marry and reproduce [13]. Men rely on their fathers and other members of the patriline to acquire brideprice for marriage [13,14]. The competition for acquisition of status and resources would be even more pronounced among unrelated men, without the mitigating effects of inclusive fitness [16]. The polygynous marriage systems that are common in Bantu societies and others with patrilineality and patrilocality lead to a skew in reproductive success, meaning that male-male competition is likely to be very intense [17].

3.2 More women will be accused of witchcraft in societies where polygynous marriage is common, compared to those where it is limited

As wives in polygynous marriages compete with each other for reproductive resources, there will be more witchcraft accusations targeting women in societies where there are higher levels of polygyny, compared to those where there are lower levels [11,12,15,18].

3.3 More men than women will be accused of witchcraft in societies with greater social stratification

Where resources are unevenly distributed and there is greater variation in male social status, there will be greater competition between men to acquire the resources they need to maximise their reproductive success [17]. Therefore nullifying competitors for resources and mates through the means of witchcraft accusations may be an adaptive strategy.

Society-level variables

All of the society-level variables used here, and included in the analysis (**Tables S2 and S4; Fig. S1**) are from Murdock's Ethnographic Atlas [2,3]. We re-coded the variables from original EA categories as shown below, with original category numbers shown in brackets.

Descent: major type (EA043)

0) Patrilineal (1)

1) Non-patrilineal (original categories duolateral (2), matrilineal (3), quasi-lineages (4), ambilineal (5), bilateral (6), mixed (7)

Marital residence with kin: prevailing pattern (EA012)

0) societies with predominantly female dispersal (avunculocal 1, patrilocal 8, virilocal 10, ambi-viri 12)

1) societies with predominantly male dispersal (avuncu-uxorilocal 3, matrilocal 5, uxorilocal 9)

2) other forms of dispersal (neolocal 6)

Class differentiation: primary (EA066)

0) Some form of class distinction (original categories wealth distinction 2, elite stratification 3, dual stratification 4)

1) Absence of wealth distinctions (original category 1)

Marital composition: monogamy and polygamy (EA009)

0) Limited polygamy (original category 2)
1) All other forms of polygamy (original categories: polygyny, sororal separate 4, polygyny, non-sororal cohabit 5, polygyny, non-sororal separate 6)

Here categories were combined to form a binary variable to differentiate between societies which had limited polygyny, and those where it is more prevalent.

4. FURTHER RESULTS: INDIVIDUAL AND SOCIETY-LEVEL

Table S2. Descriptive statistics for all predictor variables used in the model averaging process, by the sex of an individual targeted in a witchcraft accusation.

	**Female accused**	**Male accused**	**Total**
**Total**	157	266	423
**Relationship (accused-accuser)**			
Unrelated	43	156	199
Related	17	39	56
Affinal	39	12	51
NA	58	59	117
**Relationship (accused-victim)**			
Unrelated	37	113	150
Related	33	59	92
Affinal	52	22	74
NA	35	72	107
**Relationship (accused-accuser or accuser-victim)**			
Affinal	47	163	210
Unrelated	26	51	77
Related	57	17	74
NA	27	35	62
**Accused age**			

Elderly	21	21	42
Adult or child	136	245	381
**Situation**			
Fertility and relationships	41	34	75
Interpersonal	21	58	79
Wealth and status	24	89	113
Miscellaneous / Mixed	33	43	76
NA	38	42	80
**Accuser sex**			
Male	59	103	162
Female	16	21	37
NA	82	142	224
**Accuser sex / victim sex**			
Male	69	127	196
Female	23	27	50
NA	65	112	177
**Gains made by accusers**			
‘Witch’ is separated from accusers	29	66	95
Accused penalised / reconciliation with accusers	14	9	23
Accusers gain resources / political position	6	16	22

Accused suffers reputational damage	17	60	77
Accusation is dismissed	21	33	54
Accused is killed	11	8	19
NA	59	74	133
**Social stratification**			
Egalitarian	75	108	183
Wealth distinctions	53	137	190
NA	29	21	50
**Descent**			
Patrilineal	72	167	239
Non-patrilineal	85	99	184
**Post-marital residence**			
Female dispersal	127	180	307
Male dispersal	23	45	68
Other	7	41	48
**Polygamy**			
Limited polygyny	22	41	63
Other forms of polygyny	135	225	360

Table S3. Post-marital residence, and accusers/purported victims by relationship category and the sex of accused 'witches' (N=361). The first column indicates whether those accused of witchcraft were male or female. The second shows the type of relationship between the accused and their 'victims' and/or accusers (U = unrelated, R = blood relatives, A = affinal kin). The other columns show the post-marital residence patterns of societies (Avuncu = avunculocal, Uxor-avuncu = uxori-avunculocal, Matri = matrilocal, Neo = neolocal, Patri =

patrilocal, Uxo = uxorilocal, Viri = virilocal, Ambi = ambilocal). The number of societies with each type of post-marital residence is shown on the bottom row.

Sex of accused	Rel. Cat.	Avuncu (1)	Uxor-avuncu (3)	Matri (5)	Neo (6)	Patri (8)	Uxo (9)	Viri (10)	Ambi-Viri (12)	Totals
**Male**	**U**	25	1	22	36	59	3	15	2	163
	**R**	18	0	8	3	18	0	4	0	51
	**A**	4	0	2	1	9	1	0	0	17
**Female**	**U**	4	0	9	1	29	0	4	0	47
	**R**	3	0	5	0	17	0	1	0	26
	**A**	14	0	5	5	32	1	0	0	57
**No. of societies**		6	1	6	2	33	1	3	2	54

Table S4. Descent patterns, relationship categories of accusers/'victims' of witchcraft and the sex of accused 'witches'. The first column indicates whether those accused of witchcraft were male or female. The second shows the type of relationship between the accused and their 'victims' and/or accusers (U = unrelated, R = blood relatives, A = affinal kin). The other columns show the descent patterns of societies (Patri = patrilineal, Duo = duolateral, Matri = matrilineal, Quasi = quasi-lineages, Ambi = ambilocal, Bilateral = bilateral, Mixed = mixed). The number of societies with each type of descent pattern is shown on the bottom row.

Sex of accused	Rel. Cat.	Patri	Duo	Matri	Quasi	Ambi	Bilateral	Mixed	Totals
**Male**	**U**	59	9	48	1	7	1	38	163
	**R**	12	1	26	4	1	0	7	51
	**A**	9	0	7	0	0	0	1	17
**Female**	**U**	26	2	13	0	4	0	2	47
	**R**	14	1	8	0	1	0	2	26

	**A**	29	0	20	0	0	0	8	57
**No. of societies**		29	3	13	2	2	1	4	54

Table S5. Full parameters from the model-averaged multi-level logistic regression in the main text, which were conducted using *lme4* [19] and *MuMIn* [20]. The outcome variable is the sex of accused 'witches': 0) male, 1) female. Predictors are the age of an accused 'witch', the relationship category between the accused and their accuser and/or victim, and the sex of the accused's accuser and/or 'victim' and the situation ethnographers documented as causing the accusation. Significance levels: *** p < 0.001, ** p <0.01, *p <0.05.

Variable	Estimate	SE	95% CI Lower	OR	95% CI Upper	Relative Variable Importance
**Intercept**	1.38**	0.42				
**Accused age**						1.00
Ref: adult or child						
Accused age: old	1.17**	0.40	1.48	3.23	7.06	
**Relationship category**						1.00
Ref: affinal						
Unrelated	-2.43***	0.36	0.04	0.09	0.18	
Related	-1.76***	0.41	0.08	0.17	0.39	
NA	-1.55***	0.42	0.09	0.21	0.48	
**Accuser/victim sex**						0.14
Ref: male						
Female	-0.04	0.18	0.67	0.96	1.37	
NA	-0.01	0.10	0.81	0.99	1.21	
**Situation**						1.00
Ref: fertility and relationships						
Interpersonal	-0.99*	0.41	0.17	0.37	0.83	
Wealth and status	-1.10**	0.39	0.16	0.33	0.71	
Miscellaneous and mixed	0.06	0.42	0.46	1.06	2.41	
NA	-0.20	0.41	0.37	0.82	1.81	

Table S6. Results of multi-level logistic regression model averaging with individual and society-level variables. The outcome variable is the sex of an accused witch: 0) male and 1) female. The model includes the variables pertaining to individual relationships included within the main article (accused age, relationship between the accused and their accuser and/or 'victim' and the sex of the accuser and/or 'victim' and the situation ethnographers believed had produced the accusation), and the society-level variables from the EA (class distinctions, polygamy, descent and post-marital residence). Significance levels: *** p < 0.001, ** p <0.01, *p <0.05.

Variable	Estimate	SE	95% CI Lower	OR	95% CI Upper	Relative Variable Importance
**Intercept**	1.48**	0.53				
**Accused age**						1.00
Ref: adult or child						
Accused age: old	1.12**	0.40	1.40	3.08	6.75	
**Relationship category**						1.00
Ref: affinal						
Unrelated	-2.41***	0.36	0.04	0.09	0.18	
Related	-1.74***	0.41	0.08	0.18	0.39	
NA	-1.52***	0.42	0.10	0.22	0.50	
**Accuser/victim sex**						0.10
Ref: male						
Female	-0.03	0.16	0.71	0.97	1.32	
NA	-0.01	0.09	0.84	0.99	1.18	
**Situation**						1.00
Ref: fertility and relationships						
Interpersonal	-1.03*	0.41	0.16	0.36	0.80	
Wealth and status	-1.10**	0.39	0.16	0.33	0.71	
Miscellaneous and mixed	-0.03	0.43	0.42	0.97	2.25	
NA	-0.26	0.41	0.34	0.77	1.72	
**Descent**						0.28
Ref: patrilineal						
Non-patrilineal	0.06	0.22	0.69	1.06	1.64	
**Polygyny**						0.26
Ref: limited polygyny						
Other forms of polygyny	-0.03	0.28	0.56	0.97	1.67	
**Class**						0.54
Ref: egalitarian						
Wealth distinctions	-0.20	0.34	0.42	0.82	1.59	
NA						
**Post-marital residence**						0.33
Ref: female dispersal						
Male dispersal	0.04	0.29	0.59	1.04	1.84	
Other	-0.34	0.61	0.22	0.71	2.36	

Table S7. Results of model-averaged multilevel logistic regression using individual-level variables. Here we use the variables related to accusers, rather than the combined variable for accusers and victims. Significance levels: *** $p < 0.001$, ** $p < 0.01$, *$p < 0.05$.

Variable	Estimate	SE	95% CI Lower	OR	95% CI Upper	Relative Variable Importance
**Intercept**	1.50**	0.48				
**Accused age**						1.00
Ref: adult or child						
Accused age: old	1.20**	0.40	1.51	3.34	7.38	
**Relationship category - accuser**						1.00

Ref: affinal						
Unrelated	-2.60***	0.43	0.03	0.07	0.17	
Related	-2.02***	0.51	0.05	0.13	0.36	
NA	-1.60***	0.48	0.08	0.20	0.52	
**Accuser sex**						0.22
Ref: male						
Female	-0.14	0.35	0.44	0.87	1.72	
NA	-0.04	0.16	0.71	0.96	1.31	
**Situation**						1.00
Ref: fertility and relationships						
Interpersonal	-0.95*	0.40	0.17	0.39	0.85	
Wealth and status	-1.07**	0.38	0.16	0.34	0.72	
Miscellaneous and mixed	0.12	0.42	0.49	1.13	2.58	
NA	-0.20	0.41	0.37	0.82	1.82	

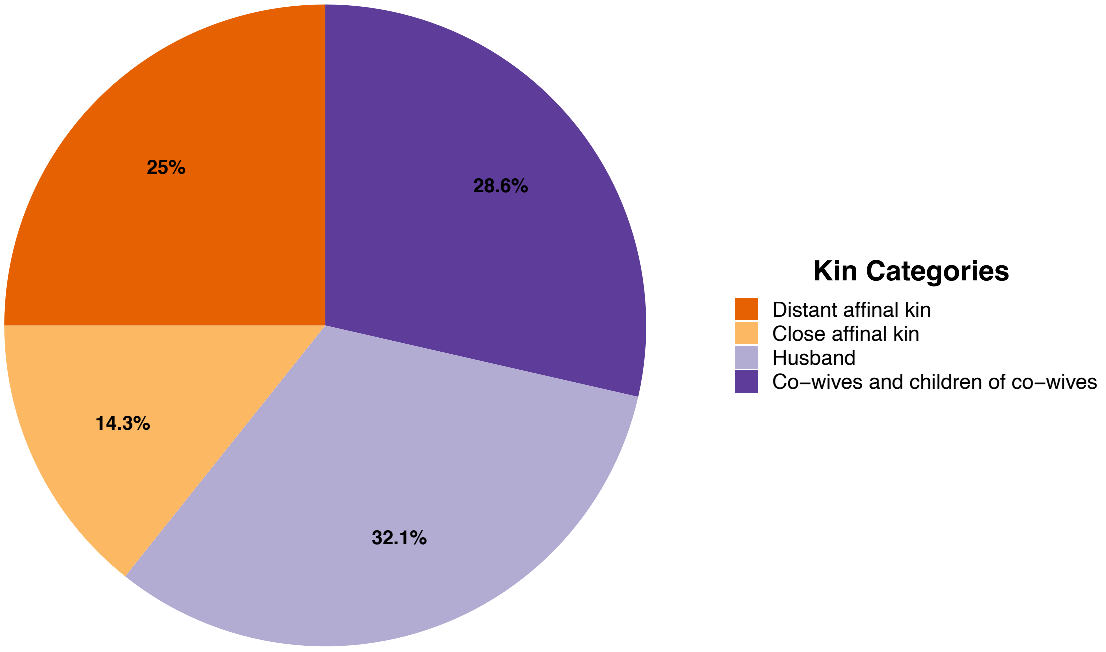

Supplementary Fig. 2. Pie chart showing the breakdown of affinal kin relationships when the target of an accusation was female (N=57). The close affinal kin category consists of mothers-in-law, fathers-in-law, sons-in-law, daughters-in-law and aunts and uncles by marriage. Distant affinal kin consists of all other categories of affinal kin.

DISCUSSION: SOCIETY-LEVEL RESULTS

None of the society-level variables significantly affected whether witchcraft accusations targeted men or women in Bantu societies. As they were all included in the top models in the selection process, they all had some effect on the outcome variable, but it was much smaller than that of individual-level variables. This does not support our predictions, previous historical and anthropological perspectives suggesting that society traits may lead to accusations being directed at a particular sex [21,22], or what might be predicted from evolutionary theory if witchcraft accusations are a mechanism for harming competitors [13,15,17].

There are several possible explanations for the lack of effect of society-level variables on the sex of individuals accused of witchcraft. The first is sample size: although this is the largest sample of its kind that we are aware of, with more detailed data, society-level differences might be more pronounced, but this was inevitably constrained by the availability of ethnographic resources.

It may also be the case that high levels of competition towards members of one sex in a society is associated with high levels of competition towards the other sex, which therefore almost cancel each other out. In patrilineal and patrilocal societies, both male-male and female-female competition is high, and there may be more inter-sexual conflict. Male-male competition is intense due to competition for mates and the resources to needed acquire them [13]. Female-female competition is strong when women are competing to obtain resources for offspring, as in societies with polygynous marriage [11,12]. Male control of females may be more pronounced where men have higher control of resources and there is a greater skew in male reproductive success [23]. The corresponding reduction in female social status could have two possible effects: it might render women less able to defend themselves and more vulnerable to being accused, but also reduced prestige and access to resources might mean accusations would be less likely to target them in the first place [24]. Because these society-level patterns of accusation may obscure and counter each other, individual-level relationships might in some ways be better at predicting competition and accusations.

It is also the case that there is limited variation between our sample of societies in some aspects of their socio-ecology. The majority of Bantu and Bantoid societies in the sample are polygynous with male-biased inheritance systems (even among matrilineal groups [2,3]), and these may be the crucial factors in determining patterns of accusation. The ICC result provides some support for this, as it suggests there was only a moderate amount of variation (15%) between societies and more substantial variation within societies in terms of the proportion of accusations directed at men and women.

Other researchers such as the historian Ronald Hutton have emphasised that finding patterns in the distribution of witchcraft beliefs proves extremely elusive, as they appear to bear little association with social organisation, environment or culture [25]. Hutton's conclusion was reached after an extensive worldwide survey of over 300 societies' witchcraft beliefs, although this was qualitative, and so may not be as precise in identifying cross-cultural patterns as quantitative methods. Hutton was examining the existence and intensity of witchcraft beliefs, rather than why accusations targeted individuals of a particular sex. But as witchcraft accusations and beliefs are highly flexible cultural traits, that can easily be adapted to a variety of circumstances, this may further account for why society-level patterns are not more pronounced.

5. Sensitivity analyses for ethnographer bias

Many ethnographers did not document witchcraft accusations systematically. Instead they mentioned cases they regarded as typical of the society at that time, or ones that illustrated a particular point, or simply those that

caught their attention. Ethnographers were more likely to report cases that were memorable or dramatic. In order to examine how much bias on the part of ethnographers may have impacted our main results, we conducted analyses using only societies where a) ethnographers attempted to systematically document all cases they heard about during a period of fieldwork and b) where ethnographers were female.

5A. Systematically documented cases

Descriptive statistics

Table S8. Counts of men and women accused of witchcraft in societies where ethnographers collected cases systematically.

**Male accused**	**Female accused**	**Total**
92	57	149
**Society *N* = 3** (Nyakyusa, Lovedu & Chewa)		

Table S9. The results given below are the same as for the main model, multilevel logistic regression using individual-level variables to predict the odds of the accused 'witch' being male or female. This is conducted on the sub-sample using cases from societies where ethnographers had attempted to document cases as systematically as possible. Significance levels: *** p < 0.001, ** p <0.01, *p <0.05.

*In this sample only one individual was categorized as old, so the 'Accused Age' variable was removed from the analysis.

**Variable**	**Estimate**	**SE**	**95% CI Lower**	**OR**	**95% CI Upper**	**Relative Variable Importance**
**Intercept**	1.72*	0.76				
**Accused age***						
**Kin category (accused-accuser)**						1.00
Ref: affinal						
Unrelated	-2.99***	0.62	0.01	0.05	0.17	
Related	-2.92***	0.78	0.01	0.05	0.25	
NA	-2.19**	0.72	0.03	0.11	0.46	
**Accuser/victim sex**						0.17
Ref: male						
Female	0.30	0.75	0.55	1.05	2.01	
NA	0.58	0.50	0.61	1.10	1.98	
**Situation**						0.65
Ref: fertility and relationships						
Interpersonal	-1.18	0.68	0.16	0.55	1.92	

Wealth and status	-0.92	0.57	0.10	0.46	2.18	
Miscellaneous and mixed	0.06	0.88	0.26	1.04	4.22	
NA	1.22	0.93	0.34	2.21	14.36	

5B. Cases documented by female ethnographers

In our sample more men than women were accused of witchcraft. This could potentially represent a bias on the part of mostly male ethnographers, who were more interested in accusations of men (although it could also be argued that European ethnographers would bring the preconception that 'witches' were female). We used a further sub-sample of all cases where an ethnographer was known to be female (two documents were produced by husband-and-wife ethnographers working together) and analysed them to see if this made difference to our results.

Descriptive statistics

Table S10. Counts of men and women accused of witchcraft when ethnographers were female.

Male accused	Female accused	Total
81	63	147
**Society *N* = 11** (Bagisu, Banen, Bemba, Chagga, Ganda, Gikuyu, Lovedu, Nyakyusa, Nyanja, Pondo, Tiv)		

Table S11. The results given below are from a multilevel logistic regression using individual-level variables to predict the odds of the accused 'witch' being male or female. This was calculated using the sub-sample of cases with female ethnographers. Significance levels: *** $p < 0.001$, ** $p < 0.01$, *$p < 0.05$.

Variable	Estimate	SE	95% CI Lower	OR	95% CI Upper	Relative Variable Importance
**Intercept**	1.29	0.78				
**Accused age**						0.25
Ref: adult or child						
Accused age: old	0.01	0.51	0.37	1.01	2.74	
**Kin category (accused-accuser)**						1.00
Ref: affinal						
Unrelated	-2.93***	0.64	0.01	0.05	0.19	
Related	-1.95*	0.02	0.03	0.14	0.71	
NA	-2.49**	0.76	0.02	0.08	0.37	
**Accuser/victim sex**						0.47
Ref: male						
Female	0.69	0.91	0.33	1.99	11.97	

NA	0.40	0.57	0.48	1.50	4.62	
**Situation**						0.42
Ref: fertility and relationships						
Interpersonal	-0.81	1.09	0.05	0.46	2.17	
Wealth and status	-0.30	0.54	0.25	0.74	2.15	
Miscellaneous and mixed	-0.04	0.58	0.31	0.96	3.02	
NA	-0.01	0.45	0.41	0.99	2.39	

In these sub-samples, the result that an accusation from affinal kin is more likely to target a woman than a man remains. Similarly, accusations from unrelated individuals have a higher odds of targeting men than women, as do accusations from blood relatives. Accusations directed at elderly people were no longer more likely to target women than men. However, this variable had to be excluded from the sub-sample of systematically recorded case studies as there was only one accusation in that category. The number of elderly individuals remained small in the sample of accusations recorded by female ethnographers ($N = 11$).

We have used these subsamples to investigate the extent that ethnographer bias may have influenced our results. The finding that accusations from affinal kin are more likely to be directed at women than men, and that accusations from non-relatives and blood relatives are more likely to target men, remains similar. The greater weight of accusations in the cases used for sensitivity analyses were directed at men. This provides some assurance that our original results are not excessively biased by anecdotal sampling or focus on cases where the accused was male on the part of male ethnographers. The variable relating to the situation ethnographers identified as causing accusations no longer produces a statistically significant result.

It should be noted that these sensitivity analyses do not have satisfactory statistical power due to reduced sample sizes [26] and can therefore only be regarded as an exploratory investigation of these factors.

6. ETHNOGRAPHY META-DATA

To further examine how likely our results were to be affected by bias, we collected meta-data on aspects of how ethnographies were produced: how long the ethnographer conducted fieldwork for, how fluently they spoke the language, when the ethnography was published, and the ethnographer's nationality. We examined how these factors and the sex of the ethnographer were associated with the main variables in our analysis. These were coded for individual ethnographies, rather than ethnographers. This was intended to be an exploratory analysis

to identify any particular trends as well as the fact that including meta-data on ethnographic materials is good practice when in this type of study [27]. But we did not have hypotheses about how bias on the part of ethnographers might lead to our results, such as whether ethnographers would be more likely to record or observe accusations of women by affinal kin than reflected the actual . In terms of the variables, we predicted that the variable linking witchcraft accusations with situations might be more unreliable, as ethnographers were attempting to infer causal associations or relying on inferences from their informants to do so. With the sex of the accused and others involved (where it was documented) and relationships, there would perhaps be a reduced margin of error as such details are generally less open to as they are mostly readily observable. But as discussed in the main text there is also the potential for selection biases in what ethnographers chose to record.

Variables

Publication date The date an ethnography was produced and published might have an effect on how prevalent particular cultural biases were prevalent at the time, and how trained ethnographers were at to be aware of their own biases [27]. Publication dates were grouped into time frames: Pre-1920, 1920-1939, 1940-1959 and 1960 onwards.

Length of time in the field Ethnographers who spent more time in the field might have a better understanding of their subjects and societies.

Language This had three categories: 1) Ethnographers had learned the language and were fluent or near-fluent 2) Ethnographers who had partially learned the language but were still reliant on interpreters 3) Ethnographers who did not learn the language.

Nationality This might influence biases held by ethnographers, by determining the prevailing theories or schools of thought they were trained in. The nationality of ethnographers had to be grouped into a number of broader categories for analysis: 1) British and American, 2) British and other 3) European 4) South African and 5) Indigenous, where the ethnographer documented their own culture.

Ethnographer type The purpose of an ethnographer's field work might influence their observations. This was divided into several categories: 1) Anthropologist, 2) Missionary, 3) Colonial Official 4) Anthropologist-Other (where the ethnographer conducted fieldwork alongside practicing another profession such as medicine. This excludes the professions mentioned in other categories).

Ethnographer sex As above, this was grouped into whether the ethnography was written by a man or whether it was written by a woman or a woman and a man.

Descriptive statistics

Table S12. Showing the individual accusations of witchcraft in our dataset alongside attributes of the ethnographic records they were obtained from: publication date, the length of time ethnographers spent in the field, how fluently ethnographers stated they spoke the language of the society they were working in, the ethnographer's nationality, their primary role and their gender.

	**Individual accusations**
**Publication date**	
Pre-1920	54
1920-1939	74
1940-1959	166
1960 +	129
**Field length (years)**	
**0.5**	4
**1**	7
**1.5**	47
**2**	151
**3**	64
**4**	58
**5 +**	44
**NA**	48
**Language**	
No	5
Partial	67
Yes	222
NA	78
**Nationality**	
British-American	147
British-Other	70
European	23
South African	80
Indigenous	8
NA	
**Ethnographer type**	
Anthropologist	282
Missionary	37
Anthropologist-Other	55
Colonial Official	16
NA	33
**Gender**	
Male	249
Female	148

Methods

We collected the data mainly from the ethnographic records used in the main study. Details were generally available in the Preface and Introduction of the documents. For some, we accessed alternative online sources of information such as alumni profiles or journal articles that provided meta-data on the production of ethnographies.

Statistical analysis

Variables with multinomial categories (Situation and Relationship) were converted to dummy variables so we could examine focal categories. We intended to perform multilevel analyses with the ethnographies as random effects, but as some texts only produced one case study this led to issues with model singularity. We therefore ran logistic regressions to compare how attributes of ethnographies were associated with outcome categories for all our main variables.

Variables were checked for collinearity using *car* [28]. As some variables were collinear we excluded one from particular models when the VIFs were over 5. For example 'Ethnographer type' was often collinear with publication date, as earlier ethnographies tended to be written by missionaries and colonial officials.

Results

Table S13. Logistic regression where predictors were attributes of ethnographers and ethnographies and the outcome variable was the sex of the accused 'witch'. This was to examine whether characteristics of the ethnography were more likely to determine whether accused individuals were male or female.

	**Sex of accused (Ref: male)**<br>**ß (SE)**
**Intercept**	0.30 (0.62)
**Publication date**	
Ref: Pre-1920	
1920-1939	-0.27 (0.49)
1940-1959	-0.84 (0.45)
1960 +	-0.65 (0.39)
**Field length**	-0.16 (0.11)
**Language**	
Ref: Yes	
Partial	1.47 (0.38)***
No	-15.27 (834.68)
**Gender**	
Ref: Female	
Male	-0.02 (0.30)

Table S14 Results from three logistic regressions where the focal category was that the relationship between the accused 'witch' and their accuser/victim was1) Affinal 2) Unrelated and 3) related. The predictors were related to aspects of ethnography production.

	**Affinal**<br>**ß (SE)**	**Unrelated**<br>**ß (SE)**	**Related**<br>**ß (SE)**
**Intercept**	-1.31 (0.89)	0.80 (0.70)	-2.33 (0.88)**
**Publication date**			
Ref: Pre-1920			
1920-1939	0.52 (0.74)	-0.43 (0.55)	0.04 (0.70)
1940-1959	0.18 (0.69)	-0.92 (0.50)	1.12 (0.58)
1960 +	0.48 (0.60)	-0.52 (0.44)	0.40 (0.52)
**Field length**	-0.14 (0.15)	0.21 (0.12)	-0.12 (0.15)
**Language**			
Ref: Yes			
Partial	1.32 (0.46)**	-0.88 (0.42)*	-0.19 (0.49)
No	-14.45 (839.96)	15.45 (824.60)	-14.09 (820.03)
**Gender**			
Ref: Female			
Male	-0.22 (0.37)	-0.69 (0.32)*	1.21 (0.40)**

Table S15 Results from 4 logistic regressions showing the effects of predictors relating to the ethnographies on outcome categories relating to the situations associated with witchcraft accusations. The Ethnographer Type variable is excluded from two analyses as it was collinear with other variables.

	**Fertility and Relationships**<br>**ß (SE)**	**Interpersonal**<br>**ß (SE)**	**Wealth and Status**<br>**ß (SE)**	**Miscellaneous and Mixed**<br>**ß (SE)**
**Intercept**	-3.48	-0.77 (1.19)	-1.13 (0.88)	-1.54 (0.90)
**Publication date**				
Ref: Pre-1920				
1920-1939	-0.24 (1.24)	1.08 (0.86)	-0.25 (0.60)	0.06 (0.56)
1940-1959	-0.97 (1.24)	1.83 (0.86)*	0.31 (0.56)	-1.13 (0.57)*
1960 +	0.68 (1.17)	1.07 (0.89)	0.64 (0.49)	-2.15 (0.51)***
**Field length**	0.56 (0.27)*	-0.66 (0.24)**	0.14 (0.14)	0.22 (0.16)
**Language**				
Ref: Yes				
Partial	-2.70 (0.70)***	-1.72 (0.62)	-0.32 (0.45)	-0.45 (0.67)
No	-14.91 (2217.65)	-17.63 (2224.66)	0.36 (1.35)	2.57 (1.56)
**Ethnographer type**				
Ref: Anthropologist-Other				
Anthropologist	0.71	1.18 (0.60)	-	-
Colonial Official	-16.59 (1085.57)	-15.17 (1150.87)	-	-
Missionary	-1.57 (1.33)	1.70 (1.10)	-	-
**Gender**				
Ref: Female				
Male	-0.74 (	0.52 (0.53)	-0.27 (0.36)	0.40 (0.45)

Table S15 Logistic regressions showing the effects of predictors relating to the ethnographies on outcome categories relating to the sex of accusers or purported victims in witchcraft accusations.

	**Accuser / 'victim' sex (ref: male) ß (SE)**
**Intercept**	0.57 (1.31)
**Publication date**	
Ref: Pre-1920	
1920-1939	0.83 (1.00)
1940-1959	-0.50 (0.94)
1960 +	0.51 (072)
**Field length**	
**Language**	
Ref: Yes	
Partial	0.96 (0.67)
No	NA
**Gender**	
Ref: Female	
Male	0.42 (0.53)

For some categories estimates and standard errors are high (in Tables S13-16). This is likely to be due to there being a relatively low number of observations for that category, such as where ethnographers were colonial officials (N=16) or where they did not speak the language at all (N=5).

Some attributes of ethnographies were significantly correlated with categories in the variables.
Only having a partial grasp of the language, as compared to being fluent or near-fluent, had significant associations with particular categories across the variable indicating the sex of the accused, the relationship driving the accusation, and the situation thought to have caused an accusation. Having a partial grasp of the language indicated that ethnographers were more likely to record accusations of women.

The 'situation' variable had the highest number of significant associations. Some of these related to the date a work was published. Publications after 1960 were significantly more likely to identify the causes of accusations as mixed or miscellaneous, rather than associating them with fertility and relationships, interpersonal reasons or wealth and status. Ethnographers whose studies were published between 1940-1959 were more likely to code interpersonal factors as the cause of accusations than any of the other categories and less likely to attribute them to miscellaneous or mixed causes.

Male ethnographers were less likely to document the relationship in an accusation as between unrelated individuals than any other category and more likely to note those between related individuals than within the unrelated or affinal categories.

Discussion

Our exploratory study of the correlations between the attributes of ethnographic records and the categories identified by ethnographers in our outcome variables indicates that there are some significant correlations. The situation connected to a witchcraft accusation had a higher number of significant associations than other variables used in our main study. This may indicate that ethnographer observations of this variable are more likely to be unreliable and should be treated with caution.

It is somewhat uncertain how much the correlations in this analysis reflect true trends in bias among ethnographers. Some results have clearer possible explanations than others: a partial grasp of the language and greater reliance on interpreters might mean that ethnographers tended to rely on their preconceptions. For example European ethnographers may have expected witches to be female and not been able to interpret information about accusations of men. Other correlations, such as male ethnographers more frequently recording the significant relationship in an accusation as between relatives and less frequently as between unrelated individuals, do not have obvious explanations in terms of ethnographer bias and may be spurious.
Bias or random error may affect ethnographers' documented observations, and this is a significant limitation when conducting research using ethnographic records.

7. SOCIETIES IN THE DATASET

Table S16. A list of the societies from which we obtained our data on witchcraft accusations.

**1. Babira**	**19. Ila**	**37. Ngombe**
**2. Bagisu**	**20. Kaguru**	**38. Ngoni**
**3. Bakongo**	**21. Kamba**	**39. Nyakyusa**
**4. Bamba**	**22. Kerewe**	**40. Nyanja**
**5. Bamileke**	**23. Kpe**	**41. Nyasa**
**6. Banen**	**24. Lakeshore Tonga**	**42. Ovimbundu**
**7. Banyoro**	**25. Lamba**	**43. Pimbwe**
**8. Bemba**	**26. Lovedu**	**44. Pondo**
**9. Buye**	**27. Lozi**	**45. Sena**
**10. Chagga**	**28. Luba**	**46. Shona**
**11. Chewa**	**29. Luchazi**	**47. Tetela**
**12. Ekoi**	**30. Luvale**	**48. Tiv**

**13. Ganda**	**31. Mambwe**	**49. Tsonga**
**14. Gikuyu**	**32. Mbuti**	**50. Tswana**
**15. Giriama**	**33. Meru**	**51. Venda**
**16. Gusii**	**34. Ndebele**	**52. Xhosa**
**17. Hadimu**	**35. Ndembu**	**53. Yao**
**18. Hehe**	**36. Ngala**	**54. Zigula**

8. REFERENCES USED IN THE DATASET

1. Schebesta, P. *My Pygmy and Negro Hosts*. (Hutchinson & Co. Ltd., 1936).
2. La Fontaine, J. Witchcraft in Bugisu. in *Witchcraft and Sorcery in East Africa* (eds. Middleton, J. & Winter, E. H.) (Routledge & Kegan Paul, 1963).
3. Wing, R. P. J. van. *Études Bakongo: Sociologie – Religion et Magie* . (Desclée deBrouwer, 1960).
4. Weeks, J. H. *Among the Primitive Bakongo*. (Seeley, Service & Co. Ltd, 1914).
5. Winter, E. H. *Bwamba: A Structural-Functional Analysis of a Patrilineal Society*. (W. Heffer and Sons Ltd., 1956).
6. Egerton, F. C. C. *African Majesty: A Record of Refuge at the Court of the King of Bangangté in the French Cameroons*. (George Routledge & Sons, Ltd. , 1938).
7. Dugast, I. *Monographie de la Tribu des Ndiki (Banen du Cameroun), tome II: Vie Sociale et Familiale*. (Institut d'Ethnologie, 1956).
8. Beattie, J. Bunyoro: An African Kingdom. in *Case Studies in Cultural Anthropology* (Holt, 1960). Available at https://ehrafworldcultures.yale.edu/document?id=fk11-002 (August 3, 2018).
9. Beattie, J. Sorcery in Bunyoro. in *Witchcraft and Sorcery in East Africa* (eds. Middleton, J. & Winter, E. H.) (Routledge & Kegan Paul, 1963). Available at https://ehrafworldcultures.yale.edu/document?id=fk11-008. (August 3, 2018).
10. Beattie, J. H. N. Divination in Bunyoro. in *Magic, Witchcraft and Curing* (ed. Middleton, J.) 211–330 (Natural History Press, 1967). Available at https://ehrafworldcultures.yale.edu/document?id=fk11-006. (August 3, 2018).
11. Brelsford, W. V. *The Succession of Bemba Chiefs: A Guide for District Officers*. (The Govt. Printer, 1944). Available at https://ehrafworldcultures.yale.edu/document?id=fq05-006. (November 11, 2018).
12. Richards, A. *Land, Labour and Diet in Northern Rhodesia*. (Oxford University Press; International African Institute, 1939).

13. Colle, R. P. *Les Baluba*. (Cyrille Van Overbergh, 1913).

14. Stahl, K. M. *History of the Chagga People of Kilimanjaro*. (Mouton, 1964).

15. Dundas, C. *Kilimanjaro and Its People: A History of the Wachagga, Their Laws, Customs and Legends, Together With Some Account of the Highest Mountain in Africa*. (H. F. & G. Witherby, 1940).

16. Natara, S. Y. *Man of Africa*. (Religious Tract Society, 1934).

17. Marwick, M. G. *Sorcery in its Social Setting*. (Manchester University Press, 1965).

18. Bystrom, K. Notes on the Ekparabong Clan. *Orient. Suec.* **3**, 1–26 (1954).

19. Talbot, A. P. *In the Shadow of the Bush*. (William Heinemann, 1912).

20. Kiwanuka, M. S. M. S. *A History of Buganda From the Foundation of the Kingdom to 1900*. (Afrikana Publishing Corporation, 1972).

21. Mair, L. P. *An African People in the Twentieth Century*. (Routledge, 1934).

22. Roscoe, J. *The Baganda*. (Cambridge University Press, 1911).

23. Routledge, W. S. *With a Prehistoric People, the Akikuyu of British East Africa: Being Some Account of the Method of Life and Mode of Thought Found Existent Among a Nation on its First Contact with a European Civilisation*. (Edward Arnold, 1910). Available at https://ehrafworldcultures.yale.edu/document?id=fl10-007. (July 30, 2018).

24. Johnstone, H. B. Notes on the customs of the tribes occupying the Mombasa Sub-District, British East Africa. *J. Anthropol. Inst. Gt. Britain Irel.* **32**, 263–272 (1902).

25. LeVine, R. A. Witchcraft and Sorcery in a Gusii Community. in *Witchcraft and Sorcery in East Africa* (eds. Middleton, J. & Winter, E. H.) (Routledge and Kegan Paul Ltd, 1963).

26. Brown, G. & Hutt, A. *Anthropology in Action: An Experiment in the Iringa District of the Iringa Province, Tanganyika Territory*. (Oxford University Press, 1935).

27. Ingrams, W. H. *Zanzibar: Its History and Its People*. (H. F. & G. Witherby, 1931).

28. Smith, E. W. & Dale, A. M. *The Ila-Speaking Peoples of Northern Rhodesia*. (Macmillan & Co., 1919). Available at https://ehrafworldcultures.yale.edu/document?id=fq06-001. (July 20, 2018).

29. Beidelman, T. O. Witchcraft in Ukaguru. in *Witchcraft and Sorcery in East Africa* (eds. Middleton, J. & Winter, E. H.) (Routledge & Kegan Paul Ltd., 1963).

30. Dundas, C. *The Organization and Laws of Some Bantu Tribes in East Africa*. *The Journal of the Royal Anthropological Institute of Great Britain and Ireland* **45,** https://about.jstor.org/terms (1915).
31. Lindblom, G. *The Akamba in British East Africa: An Ethnographical Monograph*. (K. W. Appelbergs Boktryckeri, 1916).
32. Penwill, D. J. *Kamba Customary Law: Notes Taken in the Machakos District of Kenya Colony*. (Macmillan & Co. Ltd., 1951).
33. Hurel, E. Religion et vie domestique des Bakerewe. *Anthropos* **6**, 62–94 (1911).
34. Ardener, E. *Coastal Bantu of the Cameroons (The Kpe-Mboko, Duala-Limba and Tanga-Yasa Groups of the British and French Trusteeship Territories of the Cameroons)*. (International African Institute, 1956).
35. Van Velsen, J. *The Politics of Kinship: A Study in Manipulation Among the Lakeside Tonga of Nyasaland*. (Humanities Press Inc. , 1964). Available at https://ehrafworldcultures.yale.edu/document?id=fr07-001. (August 23, 2018).
36. Doke, C. M. *The Lambas of Northern Rhodesia: A Study of Their Customs and Beliefs*. (George Harrap, 1931).
37. Krige, J. E. & Krige, J. D. *The Realm of a Rain Queen: A Study of the Pattern of Lovedu Society*. (Oxford University Press, 1943)
38. Gluckman, M. *The Judicial Process Among the Barotse of Northern Rhodesia*. (Manchester University Press, 1967). Available at https://ehrafworldcultures.yale.edu/document?id=fq09-008. (October 23, 2018).
39. Prins, G. *The Hidden Hippopotamus: Reappraisal in African History*. (Cambridge University Press, 1980). Available at https://ehrafworldcultures.yale.edu/document?id=fq09-011. (October 23, 2018).
40. Holub, E. *Seven Years in South Africa, vol. 2*. (Sampson Low, Marston, Searle and Rivington, 1895).
41. Verhulpen, E. *Baluba et Balubaises du Katanga*. (L'Avenir Belge, 1936).
42. White, C. M. N. *Elements in Luvale Beliefs and Rituals*. (Manchester University Press, 1961).
43. Watson, W. *Tribal Cohesion in a Money Economy: A Study of the Mambwe People of Zambia*. (Manchester University Press, 1958).

44. Turnbull, C. *The Forest People*. (Simon and Schuster, 1962). Available at https://ehrafworldcultures.yale.edu/document?id=fo04-003 . (July 23, 2018).

45. Balandier, G. Les villages gabonais. *Mémoires l'Institut d'Études Cent. Africaines* **5**, 1–90 (1952).

46. Moeller, A. *Les Grandes Lignes des Migrations des Bantous de la Province Oriental du Congo Belge*. (G. van Campenhout, 1936).

47. Thomas, T. M. *Eleven Years in Central South Africa*. (John Snow, 1872).

48. Turner, V. *Schism and Continuity in an African Society*. (Manchester University Press, 1957).

49. Burssens, H. *Les Peuplades de L'entre Congo-Ubangi (Ngbandi, Ngbaka, Mbandja, Ngombe et Gens d'Eau)*. vol. IV (International African Institute, 1958).

50. Weeks, J. H. *Among Congo Cannibals*. (Seeley, Service & Co. Limited, 1913).

51. Overbergh, C. van. *Les Bangala*. vol. 1 (Institut International de Bibliographie, 1907).

52. Wolfe, A. W. *In the Ngombe Tradition: Continuity and Change in the Congo*. (Northwestern University Press, 1961).

53. Barnes, J. A. *Politics in a Changing Society: a Political History of the Fort Jameson Ngoni.* (Oxford University Press, 1954).

54. Wilson, M. H. *Good Company: A Study of Nyakyusa Age-Villages*. (Oxford University Press, 1951).

55. Childs, G. M. *Umbundu Kinship and Character*. (Oxford University Press; International African Institute, 1949). Available at https://ehrafworldcultures.yale.edu/document?id=fp13-001. (August 23, 2018).

56. Edwards, A. C. *The Ovimbundu Under Two Sovereignties: A Study of Social Control and Social Change Among a People of Angola*. (Oxford University Press: International African Institute, 1962). Available at https://ehrafworldcultures.yale.edu/document?id=fp13-006 (August 23, 2018).

57. Maurice, M. Le pays de Bapimbwe. *La Geogr.* **34**, **64, 66, 67**, **68, 69, 70**. *20-31, 228-237, 309-26;* 171-189. (1935-38).

58. Hunter, M. *Reaction to Conquest: Effects of Contact With Europeans on the Pondo of South Africa*. (Oxford University Press; International African Institute, 1936).

59. Maugham, R. C. F. *Zambesia*. (John Murray, 1910).

60. Bullock, C. *The Mashona and the Matabele*. (Juta & Co., 1950). Available at https://ehrafworldcultures.yale.edu/document?id=fs05-007. (September 25, 2018).

61. Holleman, J. F. *Shona Customary Law: With Reference to Kinship, Marriage, the Family and the Estate*. (Manchester University Press, 1969). Available at https://ehrafworldcultures.yale.edu/document?id=fs05-022. (September 25, 2018).

62. Heusch, L. De. Valeur, monnaie et structuration sociale chez les Nkutshu. *Rev. l'Institut Sociol.* **1**, 73–98 (1955).

63. Akiga & East, R. *Akiga's story: the Tiv Tribe As Seen By One of its Members*. (Oxford University Press, 1939). Available at https://ehrafworldcultures.yale.edu/document?id=ff57-001. (August 20, 2018)

64. Bohannan, P. & Bohannan, L. *Tiv Economy*. (Northwestern University Press, 1968). Available at https://ehrafworldcultures.yale.edu/document?id=ff57-032. (August 20, 2018).

65. Bohannan, P. & Bohannan, L. *A Source Book on Tiv Religion*. (Human Relations Area Files. 1969. 5 v. (xv, 802 leaves) ill., 1969). Available at https://ehrafworldcultures.yale.edu/document?id=ff57-030. (August 20, 2018).

66. Junod, H. A. *The Life of a South African Tribe*. (David Nutt, 1912).

67. Schapera, L. *Married Life in an African Tribe*. (Faber & Faber Ltd., 1941).

68. Brown, T. J. *Among the Bantu Nomads*. (Seeley, Service & Co., 1926).

69. Stayt, H. A. *The Bavenda*. (Oxford University Press, 1931).

70. Wessmann, R. *The Bavenda of the Spelonken*. (African World, Ltd., 1908).

71. Soga, J.-H. *The Ama-Xhosa: Life and Customs*. (Kegan Paul, Trubner & Co. Ltd, 1931).

72. Mitchell, J. C. *The Yao Village*. (Manchester University Press, 1956).

73. Stannus, H. S. *The Wayao of Nyasaland*. (The African Department of the Peabody Museum of Harvard University, 1922).

74. Picarda. Autour du Mandera: Notes sur l'Ouzigoua, l'Oukwere et l'Oudoe. *Mission. Cathol.* **18**, 184–346 (1886).

75. Johnson, W. P. *Nyasa: The Great Water*. (Oxford University Press, 1922).

76. Taylor, B. K. *The Western Lacustrine Bantu*. (International African Institute, 1962).

77. Lambert, H. E. *Kikuyu Social and Political Institutions*. (Oxford University Press, 1956).

78. Weeks, J. H. Anthropological Notes on the Bangala of the Upper Congo River. *J. R. Anthropol. Inst. Gt. Britain Irel.* **39**, 97 (1909).

79. Werner, A. *The Natives of British Central Africa*. (Archibald Constable and Company Ltd. , 1906).

80. Colle, R. P. *Les Baluba*. (Cyrille Van Overbergh, 1913).

81. Weeks, J. H. Anthropological Notes on the Bangala of the Upper Congo River. (Part III). *J. R. Anthropol. Inst. Gt. Britain Irel.* **40**, 360–427 (1910).

82. Stannus, H. S. Notes on some tribes of British Central Africa. *J. R. Anthropol. Inst. Gt. Britain Irel.* **40**, 285–335 (1910).

6. SUPPLEMENTARY MATERIALS REFERENCES

1. Hammarström, H., Forkel, R. & Haspelmath, M. Glottolog 4.0. *Jena: Max Planck Institute for the Science of Human History* https://glottolog.org (2019).

2. Murdock, G. P. Ethnographic Atlas: a summary. *Ethnology* **6**, 109–236 (1967).

3. Kirby, K. R. *et al.* D-PLACE: A global database of cultural, linguistic and environmental diversity. *PLoS One* **11**, doi:10.1371/journal.pone.0158391 (2016).

4. Bahuchet, S. Changing Language, Remaining Pygmy. *Hum. Biol.* **84**, 11–43 (2012).

5. Turnbull, C. *Wayward Servants: The Two Worlds of the African Pygmies*. (The Natural History Press, 1965).

6. Ruhlen, M. *On the Origin of Languages: Studies in Linguistic Taxonomy*. (Stanford University Press, 1994).

7. Turnbull, C. *The Forest People*. (Simon and Schuster, 1962).

8. Beattie, J. Sorcery in Bunyoro. in *Witchcraft and Sorcery in East Africa* (eds. Middleton, J. & Winter, E. H.) (Routledge & Kegan Paul, 1963).

9. La Fontaine, J. Witchcraft in Bugisu. in *Witchcraft and Sorcery in East Africa* (eds. Middleton, J. & Winter, E. H.) (Routledge & Kegan Paul, 1963).

10. Gamer, M., Lemon, J., Fellows, I. & Singh, P. irr: Various Coefficients of Interrater Reliability and Agreement. (2019).

11. Jankowiak, W., Sudakov, M. & Wilreker, B. C. Co-wife conflict and cooperation. *Ethnology* **44**, 81–98 (2005).

12. LeVine, R. A. Witchcraft and co-wife proximity in southwestern Kenya. *Ethnology* **1**, 39–45 (1962).

13. Ji, T., Xu, J. J. & Mace, R. Intergenerational and sibling conflict under patrilocality: a model of reproductive skew applied to human kinship. *Hum. Nat.* **25**, 66–79 (2014).

14. Hartung, J. *et al.* Polygyny and Inheritance of Wealth [and Comments and Replies]. *Curr. Anthropol.* **23**, 1–12 (1982).

15. Cant, M. A. & Johnstone, R. A. Reproductive conflict and the separation of reproductive generations in humans. *Proc. Natl. Acad. Sci. U. S. A.* **105**, 5332–5336 (2008).

16. Hamilton, W. D. The genetical evolution of social behaviour. I. II. *J. Theor. Biol.* **7**, 1–52 (1964).

17. Betzig, L. L. *Despotism and Differential Reproduction: A Darwinian View of History*. (Aldine, 1986).

18. Strassmann, B. I. Polygyny as a risk factor for child mortality among the Dogon. *Curr. Anthropol.* **38**, 688–695 (1997).

19. Bates, D. M., Maechler, M., Bolker, B. & Walker, S. Fitting linear mixed-effects models using lme4. *J. Stat. Softw.* **67**, 1–48 (2010).

20. Barton, K. MuMIn: Multi-Model Inference. (2020).

21. Wilson, M. H. Witch beliefs and social structure. *Am. J. Sociol.* **56**, 307–313 (1951).

22. Nadel, S. F. Witchcraft in four African societies: an essay in comparison. *Am. Anthropol.* **54**, 18–29 (1952).

23. Hrdy, S. B. *The Woman That Never Evolved*. (Harvard University Press, 1981).

24. Thomas, T. M. *Eleven Years in Central South Africa*. (John Snow, 1872).

25. Hutton, R. *The Witch: A History of Fear, From Ancient Times to the Present*. (Yale University Press, 2017).

26. Maas, C. J. M. & Hox, J. J. Sufficient Sample Sizes for Multilevel Modeling. *Methodology* **1**, 86–92 (2005).

27. Watts, J. *et al.* Building Quantitative Cross-Cultural Databases From Ethnographic Records: Promise, Problems and Principles. *Cross-Cultural Res.* **56**, 62–94 (2022).

28. Fox, J. & Weisberg, S. *An {R} Companion to Applied Regression*. http://socserv.socsci.mcmaster.ca/jfox/Books/Companion (2019).
